# Positron emission tomography and magnetic resonance imaging methods and datasets within the Dominantly Inherited Alzheimer Network (DIAN)

**DOI:** 10.1038/s41593-023-01359-8

**Published:** 2023-07-10

**Authors:** Nicole S. McKay, Brian A. Gordon, Russ C. Hornbeck, Aylin Dincer, Shaney Flores, Sarah J. Keefe, Nelly Joseph-Mathurin, Clifford R. Jack, Robert Koeppe, Peter R. Millar, Beau M. Ances, Charles D. Chen, Alisha Daniels, Diana A. Hobbs, Kelley Jackson, Deborah Koudelis, Parinaz Massoumzadeh, Austin McCullough, Michael L. Nickels, Farzaneh Rahmani, Laura Swisher, Qing Wang, Ricardo F. Allegri, Sarah B. Berman, Adam M. Brickman, William S. Brooks, David M. Cash, Jasmeer P. Chhatwal, Gregory S. Day, Martin R. Farlow, Christian la Fougère, Nick C. Fox, Michael Fulham, Bernardino Ghetti, Neill Graff-Radford, Takeshi Ikeuchi, William Klunk, Jae-Hong Lee, Johannes Levin, Ralph Martins, Colin L. Masters, Jonathan McConathy, Hiroshi Mori, James M. Noble, Gerald Reischl, Christopher Rowe, Stephen Salloway, Raquel Sanchez-Valle, Peter R. Schofield, Hiroyuki Shimada, Mikio Shoji, Yi Su, Kazushi Suzuki, Jonathan Vöglein, Igor Yakushev, Carlos Cruchaga, Jason Hassenstab, Celeste Karch, Eric McDade, Richard J. Perrin, Chengjie Xiong, John C. Morris, Randall J. Bateman, Tammie L. S. Benzinger, Tammie L. S. Benzinger, Dominantly Inherited Alzheimer Network

**Affiliations:** 1 Washington University in St. Louis, St. Louis, MO, USA.; 2 Mayo Clinic, Rochester, MN, USA.; 3 University of Michigan, Ann Arbor, MI, USA.; 4 Institute of Neurological Research Fleni, Buenos Aires, Argentina.; 5 University of Pittsburgh, Pittsburgh, PA, USA.; 6 Columbia University Irving Medical Center, New York, NY, USA.; 7 Neuroscience Research Australia, Sydney, New South Wales, Australia.; 8 UK Dementia Research Institute at University College London, London, UK.; 9 University College London, London, UK.; 10 Massachusetts General and Brigham & Women’s Hospitals, Harvard Medical School, Boston, MA, USA.; 11 Mayo Clinic, Jacksonville, FL, USA.; 12 Indiana University School of Medicine, Bloomington, IN, USA.; 13 Department of Radiology, University of Tübingen, Tübingen, Germany.; 14 German Center for Neurodegenerative Diseases (DZNE), Tübingen, Germany.; 15 Royal Prince Alfred Hospital, Sydney, New South Wales, Australia.; 16 Niigata University, Niigata, Japan.; 17 Asan Medical Center, Seoul, South Korea.; 18 German Center for Neurodegenerative Diseases (DZNE), Munich, Germany.; 19 Edith Cowan University, Joondalup, Western Australia, Australia.; 20 University of Melbourne, Melbourne, Victoria, Australia.; 21 University of Alabama at Birmingham, Birmingham, AL, USA.; 22 Osaka City University, Osaka, Japan.; 23 Brown University. Butler Hospital, Providence, RI, USA.; 24 Alzheimer’s Disease and Other Cognitive Disorders Unit, Neurology Service, Hospital Clínic de Barcelona, IDIBAPS, University of Barcelona, Barcelona, Spain.; 25 School of Biomedical Sciences, University of New South Wales, Sydney, New South Wales, Australia.; 26 Hirosaki University, Hirosaki, Japan.; 27 Banner Alzheimer’s Institute, Phoenix, AZ, USA.; 28 University of Tokyo, Tokyo, Japan.; 29 Department of Neurology, Ludwig-Maximilians-Universität München, München, Germany.; 30 School of Medicine, Technical University of Munich, Munich, Germany.

## Abstract

The Dominantly Inherited Alzheimer Network (DIAN) is an international collaboration studying autosomal dominant Alzheimer disease (ADAD). ADAD arises from mutations occurring in three genes. Offspring from ADAD families have a 50% chance of inheriting their familial mutation, so non-carrier siblings can be recruited for comparisons in case–control studies. The age of onset in ADAD is highly predictable within families, allowing researchers to estimate an individual’s point in the disease trajectory. These characteristics allow candidate AD biomarker measurements to be reliably mapped during the preclinical phase. Although ADAD represents a small proportion of AD cases, understanding neuroimaging-based changes that occur during the preclinical period may provide insight into early disease stages of ‘sporadic’ AD also. Additionally, this study provides rich data for research in healthy aging through inclusion of the non-carrier controls. Here we introduce the neuroimaging dataset collected and describe how this resource can be used by a range of researchers.

As populations continue to age worldwide, Alzheimer disease (AD) is a pressing public health priority that requires an international response^1^. It is, therefore, unsurprising that large-scale collaborative efforts have been formed to focus on this disease. The Alzheimer Disease Neuroimaging Initiative (ADNI)^2^ and the Australian Imaging, Biomarkers and Lifestyle (AIBL)^3^ study are two of the longest-established research networks studying the progression of, and potential therapeutic treatments for, this complex disease, in cohorts with sporadic AD. The Dominantly Inherited Alzheimer Network (DIAN)^4^ brings together researchers from 21 institutions across Asia, Australia, Europe and the Americas to comprehensively and longitudinally study autosomal dominant Alzheimer disease (ADAD) carriers and their healthy non-carrier siblings.

ADAD is a very rare form of AD, accounting for ~0.01% of all cases^5^, and occurs as a result of pathogenic mutations in three genes; *APP* (amyloid-beta precursor protein), *PSEN1* (presenilin 1) and *PSEN2* (presenilin 2)^6^. These mutations are autosomal dominant, with essentially 100% penetrance; thus, offspring of individuals carrying one of these mutations have a 50% chance of inheriting it and developing AD. Mechanistically, these mutations increase amyloid-beta (Aβ) aggregation in the brain by increasing the overall production of Aβ and/or by altering the relative concentration of aggregation-prone Aβ isoforms^7^. This increase in aggregated Aβ is widely thought to be the first in a cascade of events that lead individuals to develop symptomatic ADAD, with first symptoms typically reported at 30–50 years of age^8^. More specifically, individuals carrying ADAD mutations first accumulate pathological levels of plaque-forming Aβ, followed by the formation of neurofibrillary tangles and neurodegeneration, leading to the eventual characteristic decline in cognition over a course of years to decades^5^. Given the relatively young age of those affected, age-related comorbidities are rare in ADAD, but postmortem studies do report that those dying from ADAD have higher Aβ burden and tau burden at death compared to those dying with sporadic AD^9,10^. Considering the rarity of this disease, researchers from across the globe have pooled resources to form the DIAN Observational Study (DIAN-OBS), aiming to collect longitudinal data from a large cohort of individuals with a family history of ADAD, using uniform protocols.

Beyond the utility of understanding the onset and progression of ADAD pathophysiology, there are several remarkable advantages to the longitudinal study of this disease. The relative lack of age-related comorbidities in ADAD compared to sporadic AD allows pathology to be more directly linked to biomarker and clinical changes, without the confounding influence of age. In ADAD, there is also greater certainty about the causative pathology of symptoms before autopsy, allowing for inferences to be made regarding how AD neuropathic change leads to cognitive symptoms in vivo. Furthermore, age at symptom onset in ADAD is directly linked to mutation type and is, therefore, highly predictable^8^. Unlike in sporadic AD, this phenomenon allows for individuals to be staged relative to their expected age at onset without needing to wait for an individual to become symptomatic. This allows researchers to assess pre-symptomatic changes in real time. Finally, individuals with a family history of ADAD have a 50% chance of inheriting the mutation carried by their parent. Enrolling multiple family members, including those who did not inherit a pathogenic mutation, provides well-matched controls for sibling mutation carriers specifically within the DIAN-OBS study; participation by these non-carriers also generates a rich control dataset that has potential value for studies beyond ADAD.

Leveraging the strengths of longitudinal research in ADAD cohorts, the DIAN-OBS was formed to investigate this disease using a combination of neuroimaging and other biomarker sampling methods. The unique traits of ADAD allow the DIAN-OBS design to deliver adequate power for inferences to be made while enrolling far fewer participants than would be needed to study sporadic AD. Moreover, the DIAN-OBS provides a much-needed evidence base from which trial design, including sample size estimates, can be derived. These principles are exemplified by the DIAN Trials Unit (DIAN-TU), an affiliated but separate organization established to conduct clinical trials involving ADAD family members, which recently concluded its first two drug arms^11^. Although understanding the preclinical changes that occur before cognitive symptom onset in ADAD is a critical aim of the DIAN-OBS, an additional informative potential of this study derives from the striking similarities in patterns of preclinical pathological changes that occur in both ADAD and sporadic AD^12^. For example, as in individuals with sporadic AD, the accumulation of the pathologies that occur before symptom onset conform to a characteristic temporal sequence: increased soluble Aβ, brain amyloidosis, tauopathy, brain atrophy and hypometabolism, followed by clinical and cognitive impairment^5,13,14^. Although the causative origin of these changes is known for individuals with ADAD, the mechanisms that trigger this cascade of events in sporadic AD are less clear and clearly multifactorial. Nevertheless, we propose that, in concert with the above-mentioned strengths of studying ADAD cohorts, this overlapping pathophysiology allows ADAD research to be potentially informative for the understanding of sporadic AD as well as other genetic causes of AD, such as in individuals with Down syndrome^15,16^.

Here we present an overview of the neuroimaging data that are available through the DIAN-OBS. Data acquired across this study are freely available (https://dian.wustl.edu/our-research/observational-study/dian-observational-study-investigator-resources/) and can provide researchers from a variety of fields with a large, richly phenotyped ADAD dataset that also includes data from many individuals who are healthy controls. In contrast to other AD-focused longitudinal studies, the average age of individuals within the DIAN-OBS is relatively young, making the included healthy controls an ideal data resource for longitudinal studies of younger to middle-aged individuals, who are relatively underrepresented in freely available neuroimaging datasets. To this end, the current paper aims to describe the neuroimaging data collected via this collaboration, to outline the acquisition and processing parameters and, thus, to facilitate easy access to these data for all neuroimaging researchers.

## Results

Data release 15 from the DIAN-OBS collates data from 583 individuals with a confirmed family history of ADAD. Here we describe the subset of these individuals who have completed imaging visits. The DIAN-OBS data release 15 contains imaging data for 534 participants across 205 families. Of these individuals, 23 carry mutations (*Glu693Gln* and *Ala692Gly*) that have been linked to cerebral amyloid angiopathy (CAA)^17^. For this report, participants were grouped into mutation non-carriers (*n* = 216), asymptomatic mutation carriers *(n* = 214) and symptomatic mutation carriers *(n* = 104), to evaluate data acquired in baseline imaging visits. Symptomatic status was determined using the Clinical Dementia Rating (CDR)^18^ scale, where those who scored higher than zero were considered impaired. All analyses outlined within were completed using R (version 4.2.2)^19^.

### Demographic description of baseline visit

**Fig. 1 ∣:**
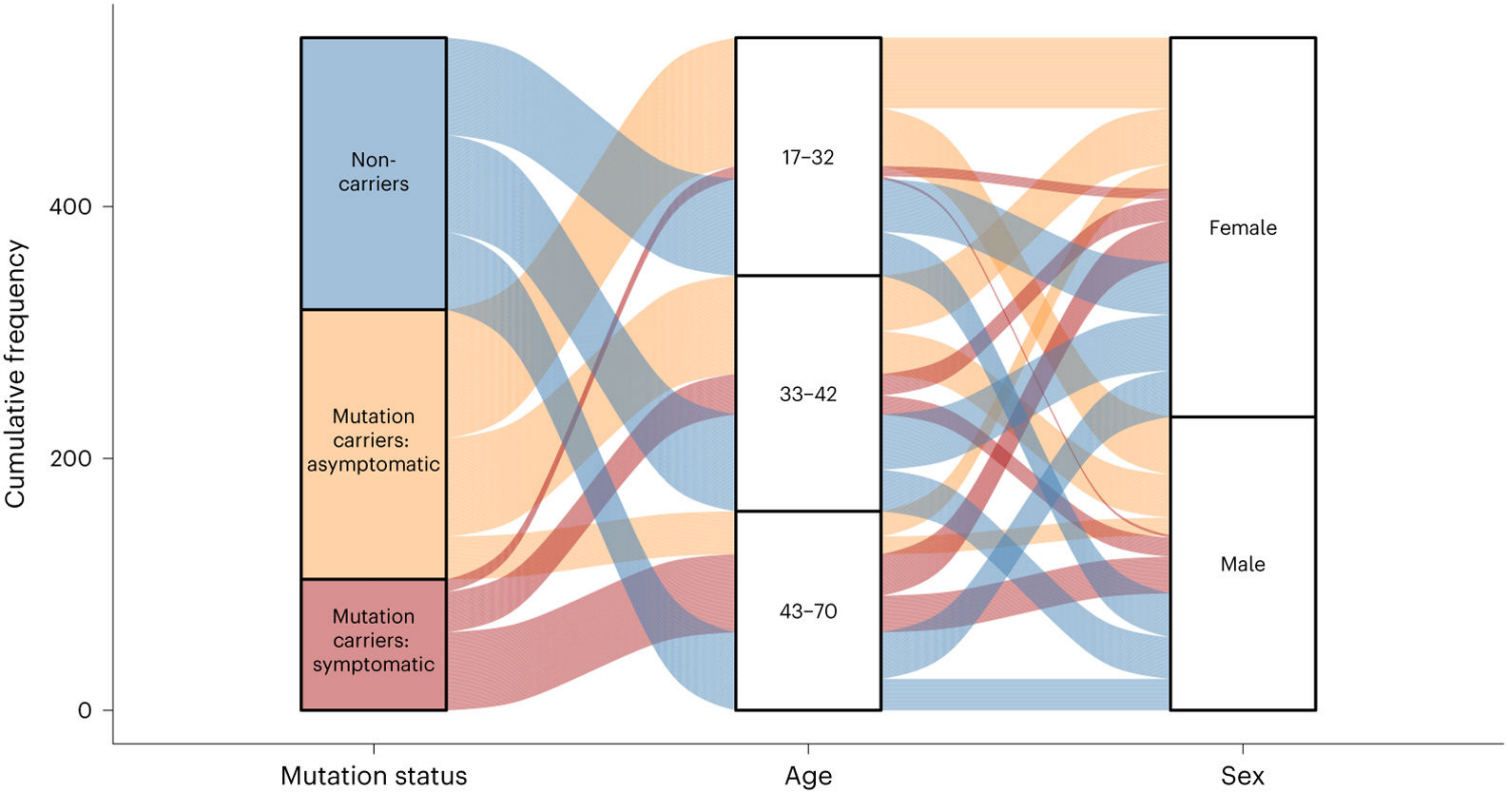
Schematic depiction of basic participant demographics for those in the DIAN-OBS with imaging data (*n* = 534). Mutation carrier and clinical status are displayed on the left panel, where each subdivision represents the proportion of participants who fall within each group (40%, 40% and 20%, respectively). The middle panel represents the proportions of total participants who fall into each age bin (M = 38.7, s.d. = 0.78), whereas the right panel represents the proportion of total participants within the DIAN-OBS who identify as female (56%) and male (44%). Colors within this plot are linked to mutation carrier and symptomatic status. These colors can be used to visually link what proportion of each panel comprises each other panel’s subgroupings. See Table 1 for further demographic information, including statistical metrics.

Members of the DIAN-OBS cohort are predominantly female (*n* = 301, 56%), right-handed (*n* = 469, 88%) and non-carriers of the *APOE* (apolipoprotein E) variant allele (*ε4*) (*n* = 378, 71%), but these characteristics are distributed in similar proportions across our three groups: males and females (χ^2^_(2)_ = 0.05, *P* = 0.97, φ = 0.01), left-handedness and right-handedness (χ^2^_(4)_ = 1.01, *P* = 0.90, φ = 0.05) and *APOE-ε4* frequencies (χ^2^_(2)_ = 0.73, *P* = 0.70, φ = 0.04). However, across these groups, there were differences in average age (*F*_(2,530)_ = 44.88, *P* = 9.88 × 10^−19^, partial η^2^ = 0.15) and average years of education (*F*_(2,530)_ = 7.56, *P* = 5.8 × 10^−4^, partial η^2^ = 0.03). More specifically, after adjusting for the influence of family and correcting for multiple comparisons (Bonferroni), symptomatic mutation carriers were of a significantly higher average age (mean (M) = 45.14, standard error (s.e.) = 0.97, 95% confidence interval (CI): 43.20–47.10) and had fewer years of education (M = 13.41, s.e. = 0.34, 95% CI: 12.90–14.10) compared to asymptomatic mutation carriers (age: M = 33.75, s.e. = 0.61, 95% CI: 32.40–35.10, *F*_(530)_ = −9.47, *P*_ADJ_ = 2.82 × 10^−19^; education: M = 14.78, s.e. = 0.19, 95% CI: 14.40–15.30, *F*_(530)_ = 3.59, *P*_ADJ_ = 1.10 × 10^−3^) and non-carriers (age: M = 37.10, s.e. = 0.75, 95% CI: 35.80–38.40, *F*_(530)_ = −6.69, *P*_ADJ_ = 1.76 × 10^−10^; education: M = 14.77, s.e. = 0.20, 95% CI: 14.30–15.10, *F*_(530)_ = 3.53, *P*_ADJ_ = 1.34 × 10^−3^). Furthermore, whereas there is no difference in years of education between non-carriers and asymptomatic mutation carriers, these groups did significantly differ in age (*F*_(530)_ = 3.47, *P*_ADJ_ = 1.71 × 10^−3^). These age and education differences likely reflect a combination of cohort effects and an artificial age division created by splitting mutation carriers by their symptomatic status. Given these differences, these two variables will be included alongside family as covariates in all remaining analyses. A full summary of these demographic details is depicted in Table 1 and illustrated in Fig. 1.

### Baseline cognitive ability of participants

Although participants completed a large number of cognitive and clinical tasks as part of the DIAN-OBS, the current paper summarizes baseline clinical and cognitive characteristics of each group using the Mini Mental State Examination (MMSE)^20^ and a general cognition composite score derived from the following tasks: the Digit Symbol task of the Wechsler Adult Intelligence Scale-Revised battery^21^, the delayed logical memory subtask of the revised Wechsler Memory Scale^22^, the Animal Naming Test^23^ and the MMSE^20^. All task scores were standardized relative to the unimpaired mutation non-carriers who were between −10 years and 0 years from the expected symptom onset, given their specific family history (*n* = 61). Scores were then averaged to derive the cognitive composite, when an individual had values for all required tests. The tests chosen for this composite index a range of cognitive abilities, including verbal fluency, executive function and declarative memory, processes that are vulnerable in early AD.

Two separate analyses of covariance models with cognition (MMSE or general score) as the independent variables, participant group as the predictor variable and age, years of education and family as covariates revealed differences in cognitive abilities between the three participant groups at their baseline study visit (MMSE *F*_(2,528)_ = 23.29, *P* = 2.03 × 10^−10^, partial η^2^ = 0.11; general score *F*_(2,528)_ = 24.22, *P* = 8.65 × 10^−11^, partial η^2^ = 0.12). After adjusting for the impact of covariates, as well as multiple comparisons, symptomatic mutation carriers had significantly poorer performance on the MMSE (M = *23.68,* s.e. = 0.95, 95% CI: 22.70–25.40) compared to asymptomatic mutation carriers (M = 29.36, s.e. = 0.32, 95% CI: 28.30–30; *F*_(529)_ = 5.93, *P*_ADJ_ = 3.36 × 10^−10^) and non-carriers (M = 29.63, s.e. = 0.45, 95% CI: 28.70–30; *F*_(529)_ = 6.58, *P*_ADJ_ = 1.60 × 10^−8^) (Table 1). Similarly, symptomatic mutation carriers had poorer average general cognition scores (M = −1.01, s.e. = 0.19, 95% CI: −1.22 to 0.71) compared to asymptomatic mutation carriers (M = 0.11, s.e. = 0.08, 95% CI: −0.11 to 0.23; *F*_(529)_ = 6.38, *P*_ADJ_ = 1.15 × 10^−9^) and non-carriers (M = 0.04, s.e. = 0.06, 95% CI: −0.14 to 0.19; *F*_(529)_ = 6.43, *P*_ADJ_ = 8.42 × 10^−10^) (Table 1). Demographic variables are visualized in Fig. 1.

### Common imaging variables of interest in AD

Analyses of baseline imaging visits revealed several structural and functional differences among the non-carriers, asymptomatic mutation carriers and symptomatic mutation carriers. Using positron emission tomography (PET), Aβ deposition and glucose metabolism were measured using [^11^C]-Pittsburgh Compound-B (PiB) and [^18^F]-fluorodeoxyglucose (FDG) tracers, respectively. A summary PiB-PET measure was computed as the average standardized uptake value ratio (SUVR) measured across the FreeSurfer Desikan atlas-derived lateral orbitofrontal, mesial orbitofrontal, rostral mesial frontal, superior frontal, superior temporal, mesial temporal and precuneus regions^24^. Using a one-way ANOVA, we observed differences in amyloid deposition (*F*_(2,478)_ = 149.15, *P* = 4.62 × 10^−51^ partial η^2^ = 0.43). Follow-up contrast revealed that symptomatic mutation carriers had significantly higher average SUVRs, signifying increased Aβ deposition (M = 2.71, s.e. = 0.13, 95% CI: 2.46–2.76) compared to asymptomatic mutation carriers (M = 1.57, s.e. = 0.05, 95% CI: 1.52–1.71; *F*_(479)_ = −10.5, *P*_ADJ_ = 5.43 × 10^−23^) and to non-carriers (M = 1.06, s.e. = 0.01, 95% CI: 0.96–1.15; *F*_(529)_ = −17.0, *P*_ADJ_ = 9.67 × 10^−51^), after correcting for partial volume effects and using a Bonferroni adjustment for multiple comparisons. There was also a significant difference in PiB uptake between non-carriers and asymptomatic mutation carriers (*F*_(529)_ = −8.28, *P*_ADJ_ = 3.65 × 10^−15^).

**Fig. 2 ∣:**
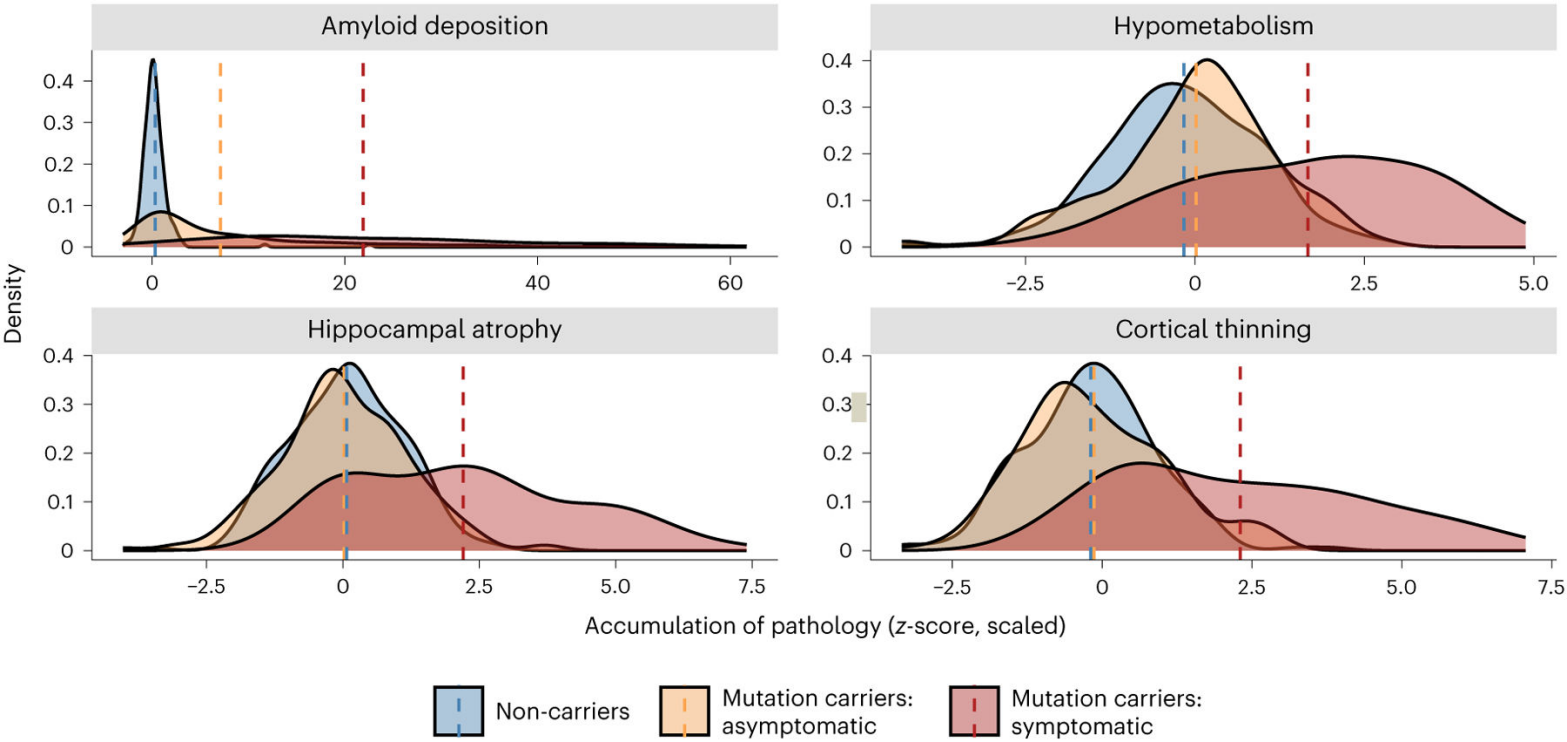
Summary depiction of the results of the analyses performed on the PiB-PET (*n* = 484), FDG-PET (*n* = 494) and T1w MRI (*n* = 534) images. Distributions represent the average accumulation of each major pathology that is present across groups at baseline visit. All *z*-scores were calculated relative to unimpaired mutation non-carriers. Dashed lines represent mean scores. No statistical tests are depicted, but a full breakdown of differences in these metrics is presented in the Results as well as in Extended Data Table 1 and Extended Data Fig. 2. In all cases, these distributions suggest that symptomatic mutation carriers (red) have higher levels of pathology than asymptomatic carriers (yellow) and non-carriers (blue). Aβ deposition represents averaged PiB-SUVR extracted from the lateral orbitofrontal, mesial orbitofrontal, rostral mesial frontal, superior frontal, superior temporal, mesial temporal and precuneus; hypometabolism represents the average FDG-SUVR extracted from the isthmus cingulate and inferior parietal regions; cortical thinning was derived from cortical thickness values averaged across the lateral orbitofrontal, mesial orbitofrontal, rostral mesial frontal, superior frontal, superior temporal, mesial temporal and precuneus; and hippocampal atrophy represents average hippocampal volume. Plot demographics: average age = 38.7 years; proportion females = 56%.

**Fig. 3 ∣:**
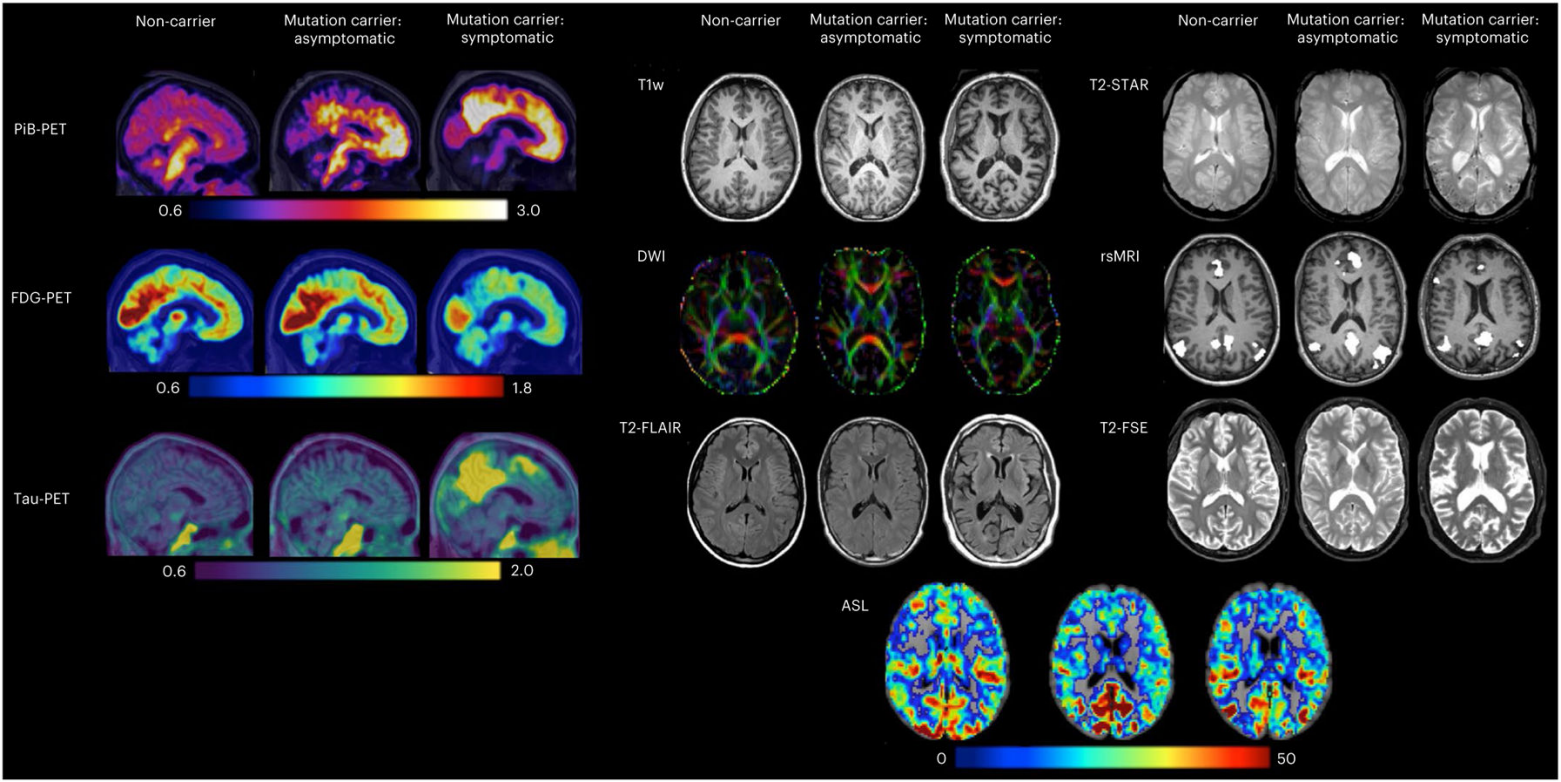
Example images representing each of the PET and MRI modalities across the DIAN-OBS. These images are depictions of a representative from each of our participant groups: non-carriers, mutation carriers–asymptomatic and mutation carriers–symptomatic (*n* = 3). In PiB-PET images, increases in uptake of the PiB tracer reflect increased amyloid deposits. In FDGPET, decreases in tracer uptake are indicative of reduced glucose metabolism (hypometabolism). For tau-PET imaging, increases in tracer uptake represent increases in tau deposits. Together, these three PET measures show greater AD pathology (amyloid deposits, hypometabolism and tau deposits) in this symptomatic mutation carrier compared to the asymptomatic mutation carrier and the control. In contrast, our MRI protocols do not measure tracer uptake, with each MR modality tailored to provide information about specific brain structures or function. T1w images are collected to assess structural morphometry, such as gray matter thickness and volume. Of note, our symptomatic mutation carrier appears to have larger ventricles and greater atrophy of the cortical ribbon compared to our representative asymptomatic mutation carrier and control. DWIs are collected to assess white matter integrity. Red, green and blue colors depict the primary direction of fiber orientation within each voxel, allowing assessment of microstructural changes in white matter. T2-FLAIR images are collected to assess white matter hyperintensities and edema. Here, several bright-white lesions can be visualized in the symptomatic mutation carrier. T2-Star, or susceptibility-weighted images, can be used to evaluate hemorrhagic lesions, such as the presence and location of cerebral microbleeds, which are common in ADAD. These can be visualized in the example symptomatic mutation carrier as small black dots. rsMRI is derived from functional MRI scans and can be used to measure the integrity of functional brain networks. These are thought to be disrupted in association with ADAD disease stage. Here, the default mode network is represented, revealing fewer regions of highly coherent activity fluctuations in the symptomatic mutation carrier. T2-FSE images are collected to quickly assess large deviations from expected structural morphometry (that is, tumors). Finally, ASL images are collected to assess cerebral perfusion. Deriving maps of perfusion allow quantification of the rate of cerebral blood flow, which is thought to decrease as a function of ADAD disease stage.

Similarly, we report differences in the average FDG-PET-derived SUVR across the FreeSurfer Desikan atlas-derived isthmus cingulate and inferior parietal regions (*F*_(2,488)_ = 51.44, *P* = 5.34 × 10^−21^, partial η^2^ = 0.23). Subsequent follow-up contrasts showed that symptomatic mutation carriers have significantly lower SUVR (M = 1.47, s.e. = 0.02, 95% CI: 1.46–1.53), indicating lower levels of glucose metabolism compared to asymptomatic mutation carriers (M = 1.68, s.e. = 0.01, 95% CI: 1.65–1.70; *F*_(489)_ = 8.22, *P*_ADJ_ = 5.60 × 10^−15^) and non-carriers (M = 1.71, s.e. = 0.01, 95% CI: 1.69–1.73; *F*_(489)_ = 10.0, *P*_ADJ_ = 3.04 × 10^−21^), after correcting for partial volume effects and using a Bonferroni adjustment for multiple comparisons. There were no differences in SUVR between non-carriers and asymptomatic mutation carriers (Extended Data Table 1 and Figs. 2 and 3).

Finally, two independent ANOVAs of T1-weighted (T1w) magnetic resonance imaging (MRI) data pre-processed using FreeSurfer (version 5.3 (ref. 25)) revealed significant differences in hippocampal volume (*F*_(2,528)_ = 75.89, *P* = 1.07 × 10^−29^, partial η^2^ = 0.30) and cortical thickness (*F*_(2,528)_ = 89.32, *P* = 2.53 × 10^−1^, partial η^2^ = 0.36). Follow-up two-tailed contrasts showed that symptomatic mutation carriers have significantly smaller total hippocampal volumes, after accounting for intracranial volume and covariates, recorded at their baseline visit (M = 7,440.18, s.e. = 132.84, 95% CI: 7,398–7,754) compared to asymptomatic mutation carriers (M = 8,881.26, s.e. = 53.37, 95% CI: 8,700–8,939; *F*_(529)_ = 11.0, *P*_ADJ_ = 4.86× 10^−25^) and non-carriers (M = 8848.97, s.e. = 45.57, 95% CI: 8,729–8,961; *F*_(529)_ = 11.7, *P*_ADJ_ = 1.15 × 10^−25^), after using a Bonferroni adjustment for multiple comparisons. No difference for hippocampal volume was found between asymptomatic mutation carriers and non-carriers. Furthermore, the cortical thickness measure used an AD-specific signature mask of the left isthmus cingulate, the left and right precuneus and right hemisphere inferior parietal, superior parietal and lateral occipital regions^26^. The follow-up pairwise two-tailed contrasts found decreased total thickness in symptomatic mutation carriers (M = 2.07, s.e. = 0.02, 95% CI: 2.08–2.13) compared to asymptomatic mutation carriers (M = 2.33, s.e. = 0.01, 95% CI: 2.30–2.33; *F*_(529)_ = 11.4, *P*_ADJ_ = 1.42 × 10^−33^) and non-carriers (M = 2.34, s.e. = 0.01, 95% CI: 2.32–2.36; *F*_(529)_ = 13.0, *P*_ADJ_ = 2.51 × 10^−26^), after using a Bonferroni adjustment for multiple comparisons. No difference for cortical thickness was found between asymptomatic mutation carriers and non-carriers (Extended Data Table 1 and Extended Data Figs. 2 and 3).

### Scan availability within the DIAN-OBS

Individuals completed three separate sessions of baseline neuroimaging scans to cover: MRI, PiB-PET and FDG-PET. Given that some individuals did not complete all sessions, or their scans might have failed quality control assessments, Table 2 summarizes the number of usable scans that are available within the DIAN-OBS data release 15 while also outlining the numbers of individuals who have completed multiple imaging visits. Note that, although tau-PET scans are not available in the current DIAN-OBS data release 15, they are currently being collected and processed in preparation for future DIAN-OBS data releases. In the coming year, we expect the number of acquired tau-PET images to greatly increase.

#### Key variables and recommendations for using DIAN-OBS data Estimated year of symptom onset.

**Fig. 4 ∣:**
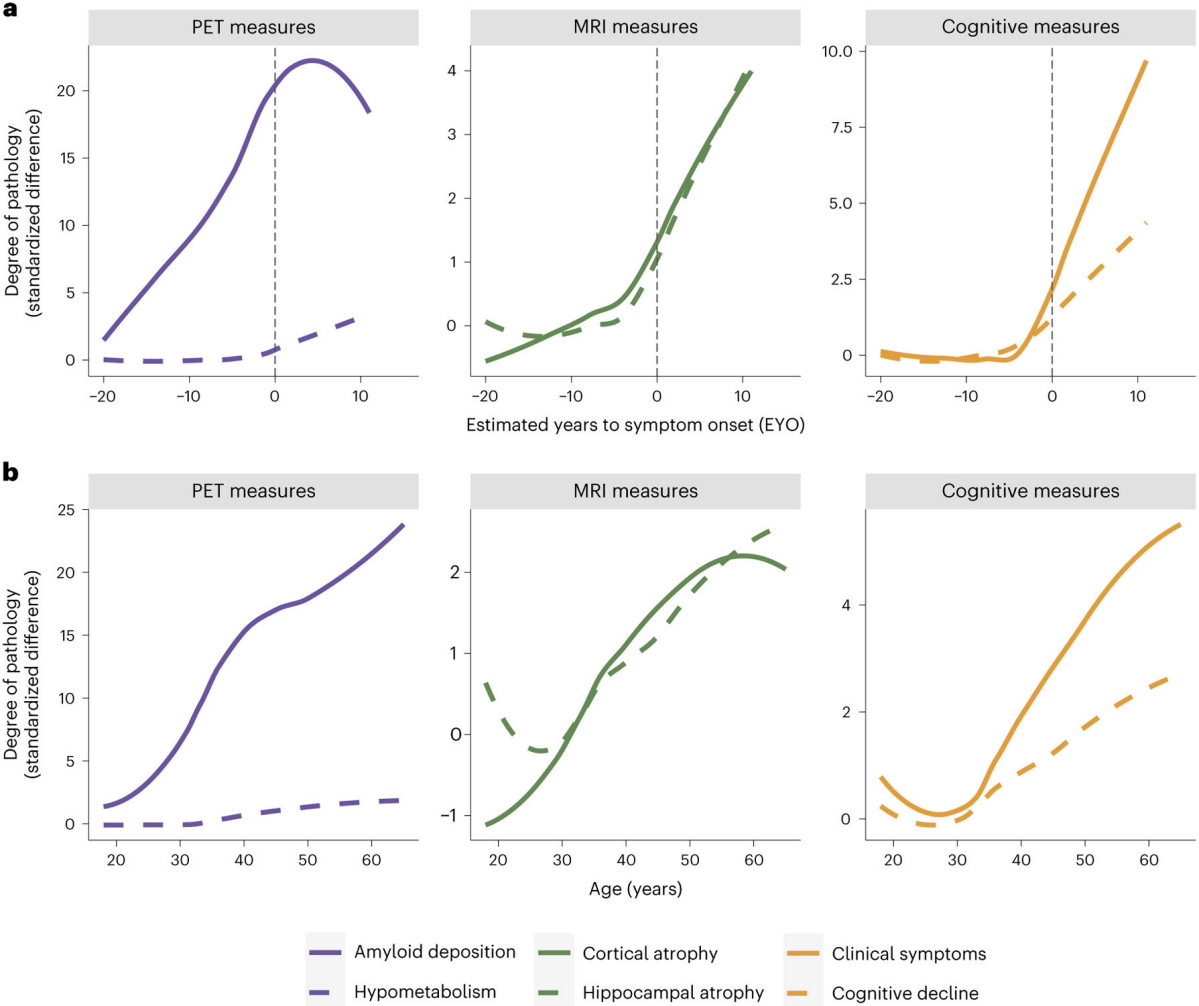
Visualization of the utility of using the EYO variable in DIAN-OBS analyses. **a**, Curves represent levels of baseline biomarkers for mutation carriers (*n* = 318), relative to non-carrier controls (*n* = 216). These are plotted against EYO to depict how levels of these markers increase relative to symptom onset (black dashed line), in those who are mutation carriers. Aβ accumulation begins many years before EYO, whereas neurodegenerative pathology occurs much closer to the EYO timepoint. Of note is the onset of clinical and cognitive symptoms that appear right before EYO. In comparison, **b** aligns these same values by participants’ ages. When assessing the trajectories using age, the relative temporal ordering of these biomarkers is obscured. In the case of ADAD, age is not a good proxy for disease stage. Plot demographics: *n* = 318, average age = 39.4 years, proportion of females = 56%.

The highly predictable age of cognitive symptom onset allows researchers to stage mutation-carrying individuals relative to their estimated year of symptom onset (EYO)^5^. It is possible to estimate EYO based upon the individual’s point mutation, the average age of onset for the gene that their mutation is linked to or the age of symptom onset for their affected parent. Although all these individual estimates are available in the DIAN-OBS release, a unique EYO is also released based on the joint consideration of all three of these aspects. In this case, EYO is presented as a number, representing years, where zero is the estimated point of conversion to symptomatic status, negative numbers indicate the time until conversion and positive numbers indicate the years since conversion. In mutation carriers, EYO is also updated upon reaching symptomatic status to ensure accuracy. The full utility of considering EYO is clearly demonstrated when examining cross-sectional data within the DIAN-OBS (Fig. 4a). The ability to stage individuals relative to their conversion point is not possible in preclinical sporadic AD cohorts without waiting years to confirm whether cognitively unimpaired participants develop AD. However, when visualizing pathology accumulation using EYO, a clear temporal pattern emerges describing the magnitude and order of changes that occur in the progression of ADAD, marked by Aβ deposition in the first stage, followed later by atrophy and hypometabolism and, finally, cognitive decline^5^. In contrast, the same cross-sectional data aligned by age does not show such a striking pattern, clearly demonstrating that, unlike EYO, age is not a useful proxy of disease stage across the entire DIAN-OBS cohort (Fig. 4b).

#### Genetic information.

Participants in the DIAN-OBS are genotyped to confirm their ADAD mutation status as well as sequenced for commonly investigated single-nucleotide polymorphisms (SNPs). Using this information, DIAN-OBS participants are classified as mutation carriers or non-carrying controls. Commonly, researchers will combine this classification with an individual’s CDR value to split mutation carriers into those who are asymptomatic and symptomatic. This allows researchers to compare mutation carriers in the preclinical (that is, asymptomatic) phase of ADAD to those in the symptomatic phase. Researchers also commonly categorize individuals by the gene that their ADAD mutation is linked to–*PSEN1, PSEN2* or *APP* (Extended Data Fig. 2)–recognizing that each of these genes influences the abnormal accumulation of Aβ via a similar, but distinct, biological process^6,27^. It is important to note, however, that even within the *PSEN1* group of ADAD mutations, there is heterogeneity in the phenotypic expression of cortical Aβ, related to the specific location of the affected codon^27^. Thus, this ADAD gene-based approach to categorization might benefit from greater refinement.

Within the DIAN-OBS sample, 23 individuals carry mutation variants (*Glu693Gln* and *Ala692Gly*) that have been linked to CAA, a disease that is distinct from ADAD^17^. These mutations occur within the *APP* gene and are associated with cerebral hemorrhages both in the presence (*Ala692Gly*) and absence (*Glu693Gln*) of AD pathology. Although both ADAD and CAA pathologies are driven by impaired Aβ clearance, the mechanisms underlying their phenotypic expression are unique^28^.

In addition to considering ADAD mutations, many researchers are interested in the impact of common variants in genes coding for proteins such as APOE and brain-derived neurotrophic factor (BDNF). SNPs in these genes have previously been linked to sporadic AD and represent secondary genetic influences that may have a modifying impact on the accumulation of AD pathology alongside ADAD mutations. Studying the impact of these alleles in sporadic AD can be extremely complicated, given the multifaceted causes of this form of AD. However, the precise timing of symptom onset inherent for ADAD mutations makes it possible for researchers to use comprehensive genetic data to test hypotheses about how secondary factors moderate the onset of Aβ accumulation or other early preclinical AD changes.

#### Important covariates to consider.

As noted, age and years of education do significantly differ among the non-carrying controls, asymptomatic mutation carriers and symptomatic mutation carriers of the DIAN-OBS cohort (Table 1). When using age in analyses, it is important to recognize that age and EYO are both confounded with symptomatic status within the mutation carriers, as the asymptomatic stage by nature must temporally precedes the symptomatic stage. Because both EYO and age are time dependent, they explain overlapping variance within data. As previously demonstrated (Fig. 4), EYO is a more precise indicator of disease progression, given that it tightly aligns with disease stage, whereas age does not. It is for this reason that we do not recommend using age and EYO in the same models.

Given the family structure of the individuals within the DIAN cohort, it is also important that researchers include family identification as a covariate in analyses to account for shared variance arising from a family’s shared cultural, genetic and environmental backgrounds. By incorporating this clustered population structure, researchers can draw stronger inferences from their statistical results, by reducing errors associated with regression coefficient estimates.

Finally, a major challenge of multi-site data collection is the management of hardware-dependent variance. This noise is introduced when multiple scanner types are included for data acquisition across the various global sites. Although the DIAN-OBS study has endeavored to minimize this variance by employing a small number of distinct scanner models and implementing unified quality control and pre-processing measures, it may be important for researchers to control for differing scanner models within their analyses.

#### Using data from the DIAN-OBS for research beyond AD.

Beyond its clear utility for understanding the development of ADAD, the DIAN-OBS enrolls cognitively unimpaired mutation non-carriers. Given the rich phenotypic data associated with each study visit, this subsample of the DIAN cohort is an ideal resource for understanding a wide range of biological processes in healthy individuals. Additionally, the longitudinal nature of the DIAN-OBS facilitates these data being useful for monitoring changes in processes across the adult lifespan. These data also represent a diverse range of individuals sourced from centers around the globe and span a middle age range that is relatively underrepresented in open neuroimaging datasets, which primarily consist of younger and/or older adults^29^. A summary visualization of key demographic variables of interest for these individuals is depicted in Extended Data Fig. 3.

Recent studies have used the DIAN-OBS dataset to supplement more commonly available samples of younger adult and older adult controls to form adult lifespan datasets^30-33^. Such datasets might be useful in examining healthy age differences continuously across the adult lifespan and to train normative machine learning models to predict biological age^34^. Indeed, the extensive data collected for each participant make the DIAN-OBS a truly unique and valuable data resource, even when considering only the control subsample. For example, the DIAN control cohort was fundamental in the discovery of large age-related increases in cerebrospinal fluid (CSF) neurofilament light chain levels, previously thought to be a strong marker of disease^35^. Given that the DIAN control cohort is richly phenotyped with imaging, genetic, cognitive, biofluid and clinical samples, data from these individuals can be used as a baseline reference for answering a variety of research questions.

## Discussion

**Fig. 5 ∣:**
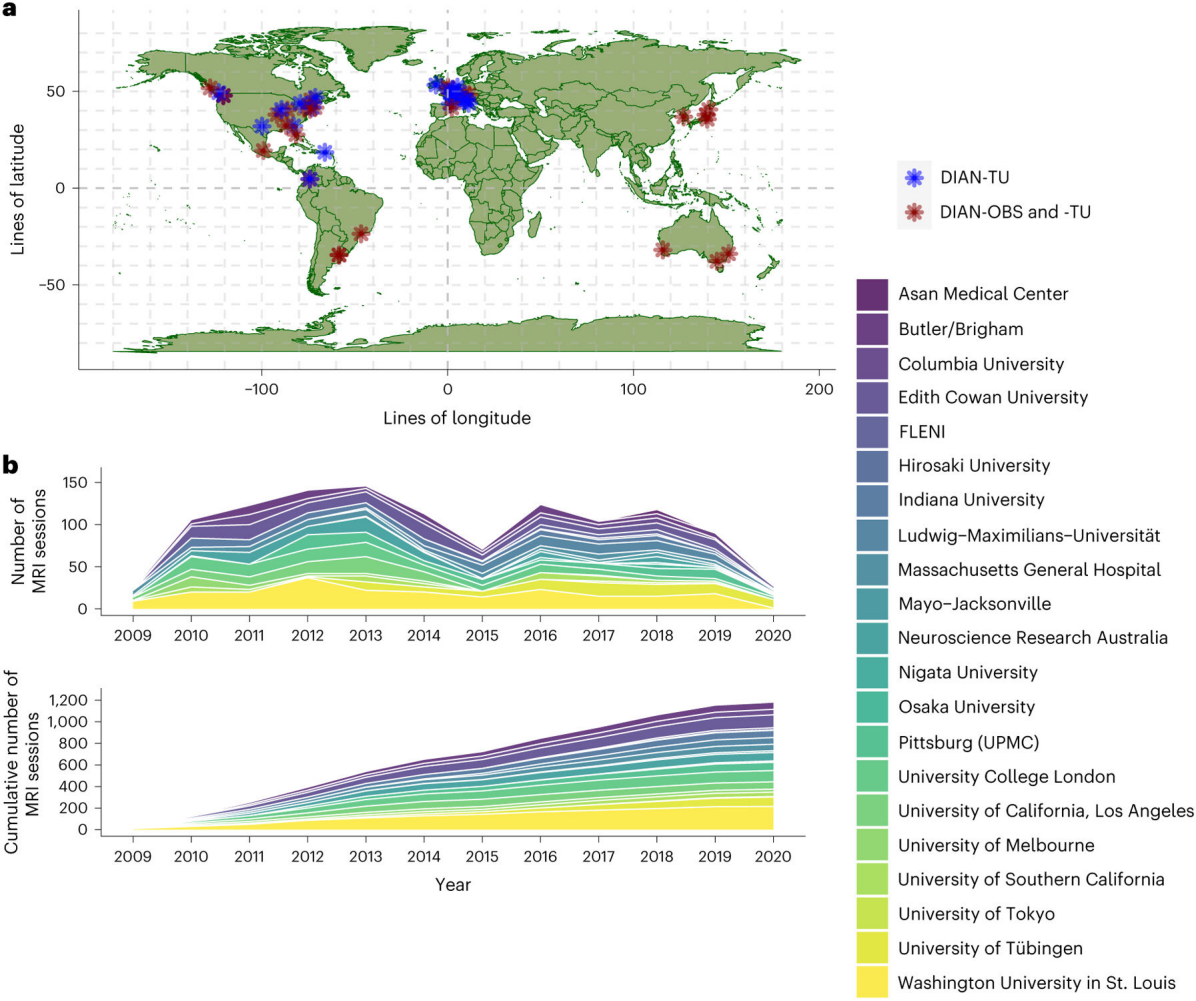
Global contributions to the DIAN-OBS and DIAN-TU studies. **a**, Depiction of the global network of researchers collecting DIAN-affiliated data. Blue indicates sites that are only involved in the DIAN-TU, whereas red indicates sites that are involved in both the DIAN-TU and DIAN-OBS. **b**, A stacked area plot depicting the relative contributions to imaging data for each of the DIAN-OBS sites. This plot further illustrates the evolution of the DIAN-OBS as a global study, originally beginning with only 10 sites and growing to 21 active sites.

**Fig. 6 ∣:**
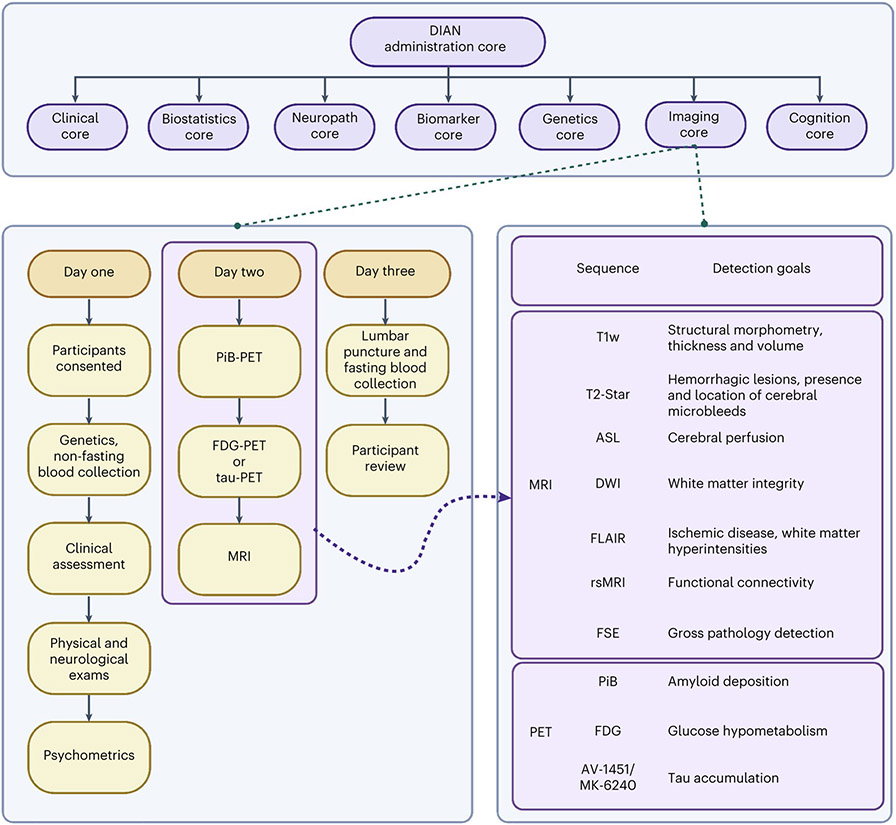
An overall schematic of the DIAN-OBS study and the contributions of the Imaging Core. The DIAN-OBS comprises seven core centers, each working symbiotically to collect, analyze and prepare data for regular data releases. In the original grant cycle, these visits were completed every 3 years for asymptomatic participants and annually for symptomatic participants. After the DIAN grant cycle was renewed, the visit frequency was updated to be completed every 2 years for asymptomatic participants and annually for symptomatic participants. Given that most participants live large distances from the study sites, they typically travel to the location and complete all parts across 3 d. If1110.1038/s41593-023-01359-8participants live large distances from the study sites, they typically travel to the location and complete all parts across 3 d. If participants live in a study site city, they are able to complete the various components of the visit within 3 months. As the DIAN-OBS begins to implement tau-PET scans at applicable sites, these will be collected in place of the FDG-PET scans. Asymptomatic non-carriers stop in-person visits 5 years after they surpass their parental age of symptom onset. Figure created with BioRender.com.

The DIAN-OBS contributes to our growing understanding of ADAD by facilitating the global study of individuals from families with a known history of this rare disease (Fig. 5). Given that age at symptom onset is highly heritable within families and for individual mutations, the DIAN-OBS uniquely allows researchers to study the preclinical phases of ADAD by staging participants based on their EYO. The relatively young age at symptom onset and the known causative mechanism of disease pathology are inherent strengths of studying ADAD cohorts. Individuals who do not inherit their family’s mutation are also included in the DIAN-OBS as well-matched controls. These individuals may also serve as a well-characterized control sample for research outside of ADAD. Together, the unique strengths of studying ADAD, along with the rich data collected across many modalities, allow the DIAN-OBS to investigate aspects of ADAD with higher confidence than has been possible for the study of sporadic AD. To that end, over 200 studies have been published using DIAN-OBS data, highlighting the tremendous contribution that these data have already had on our scientific understanding of ADAD. Data acquired as part of the DIAN-OBS are freely available upon request and represent the first resource of this magnitude, covering a diverse range of ADAD biomarker data. Here, we outline the extensive neuroimaging data available as part of this study, offering insight into the acquisition of this data, and its vast utility (Fig. 6).

Data derived from the DIAN-OBS have been critical for understanding the temporal order of pathological changes that occur in the two decades before cognitive symptom onset in ADAD. By aligning data using EYO for the cohort’s mutation carriers, a clear evolution of pathology has been demonstrated, showing that Aβ pathology occurs first, followed by tauopathy, neurodegeneration and hypometabolism and eventual cognitive decline^5^. Although this seminal work was conducted using data from individuals with ADAD, an identical hierarchy of biomarker changes is observed in cohorts representing the development of sporadic AD^12^. Notably, the biological mechanisms that underpin these two forms of AD are distinct. Although ADAD is the direct outcome of genetically determined increases in pathological Aβ, the etiology of sporadic AD is multifaceted, likely the result of combined lifestyle, genetic and environmental factors. Despite this, the cascade of events that directly precede the onset of cognitive decline for these distinct groups is extremely similar^12^. Leveraging this similarity allows researchers to posit that ADAD is informative for understanding other forms of AD, and this shared pathobiological construct suggests that mechanism-based interventions successful in ADAD could be used to treat other forms of AD. Notably, the known causative nature of the ADAD mutations further allows researchers to observe the preclinical period of AD in individuals without having to wait for them to become symptomatic or having to enroll very large numbers of participants, as would be the case in sporadic AD. These inherent strengths of studying ADAD cohorts position the DIAN-OBS as a potential model for testing theories regarding the moderating impacts of secondary influences on the trajectories of AD outcomes.

Despite similarities in the hierarchical ordering of ADAD and sporadic AD pathologies, DIAN-OBS neuroimaging data have been fundamental for understanding differences in the phenotypic expression of these two disease forms. Using data from this study, researchers have uncovered differences in the spatial patterns of pathological accumulation as well as differences in the magnitude of AD-associated pathologies in ADAD, compared to sporadic AD. More specifically, pathology associated with Aβ, tau, hypometabolism and atrophy is first reported in the precuneus, a structure that remains one of the most prominently impacted regions across the disease course of ADAD^13,14,36,37^. In contrast, the medial temporal cortex is most associated with pathology in sporadic AD. In line with prior neuropathological work, MRI and PET imaging have also revealed that regions such as the thalamus and putamen, not typically associated with sporadic AD, are common sites of ADAD-related pathologies^36^. Tau-PET imaging recently observed that, compared to sporadic AD, ADAD is associated with a much higher ratio of cortical to subcortical tauopathy^14^. Vascular-related pathologies, such as cerebral microbleeds and white matter hyperintensities, also show distinct patterns in this group^38-40^. There are also reports of Lewy body and transactive DNA-binding protein 43 (TDP-43) proteinopathies that are unrelated to age in ADAD^41^. Functional MRI analyses have revealed that ADAD is associated with a preferential degradation of cognitive networks over sensorimotor networks, a selective pattern consistent with sporadic AD and distinct from healthy age differences^42-45^. Finally, neuroimaging studies have indicated that the burden of these pathologies appears to be larger in individuals with ADAD, although the rate of accumulation is not unform across the brain^13,37^. Postmortem studies of ADAD have independently confirmed these unique spatial patterns as well as increased tau burden and Aβ burden in these individuals ^9,10^. Such data collected via the DIAN-OBS have greatly extended our understanding of the similarities and differences in the patterns of Aβ accumulation, tauopathy, hypometabolism and neurodegeneration in these forms of AD^36,37^.

Critically, the DIAN-OBS has provided the scientific framework behind the formation of the DIAN-TU^46^. Beginning in 2012, the DIAN-TU was one of the world’s first collaborations focusing on the prevention of ADAD. The DIAN-TU enrolls individuals from families who carry ADAD genetic mutations, many of whom may have participated in the DIAN-OBS. The main goal of the DIAN-TU is to assess the safety, tolerability and effectiveness of drugs that may improve the lives of those at risk of, or living with, AD. These studies will help researchers understand whether these drugs can be used to prevent, delay or even reverse the neuropathological changes that occur in ADAD. The DIAN-TU enrolled 194 (ref. 11) individuals from across the globe in its first two drug arms assessing drugs targeting Aβ. The DIAN-TU will also enroll 168 individuals from 40 sites across 16 countries in an upcoming drug arm targeting both Aβ and tau deposition with the brain (ClinicalTrials.gov identifier: NCT05269394). The DIAN-OBS was fundamental for the inception of these trials, as it allowed for researchers to demonstrate the statistically powerful design inherent in this cohort. The precise timing that can be inferred using EYO allows the DIAN-TU to precisely monitor the impact of primary and secondary mechanisms of influence in a controlled environment. Finally, the importance of the DIAN-TU is underscored by recent work suggesting that ADAD shares consistent pathophysiology patterns with sporadic AD^12^.

Moving forward, the recent DIAN grant renewal explicitly focused on elucidating the impact of ADAD mutations on proteoform signatures of Aβ and tau and the subsequent neuroinflammatory response that they elicit as well as patterns of neuronal and synaptic injury that emerge across the disease course. To accomplish these aims, neuroimaging will be used in conjunction with mass spectrometry and other molecular techniques to quantify the presence and amount of each protein isoform, establishing their relationship with PET-measured Aβ and tau in vivo^47^, in postmortem brain tissue^9,10^ and in ADAD mutation-derived pluripotent stem cells^48^. The expanding richness of the DIAN-OBS data is also reflected in the targeted proteomic approach to understanding ADAD-specific neuroinflammation and neurodegeneration, resulting in quantification of proteins such as neurofilament light chain, chitinase-3-like protein 1/YKL-40, visinin-like protein-1 (VLIP-1) and synaptosomal-associated protein 25 (ref. 49). In line with these grant aims, many ongoing DIAN-affiliated projects further focus on understanding phenotypic heterogeneity that arises from ADAD mutations. For example, recent work has highlighted that the location of ADAD mutations along *PSEN1* modulates Aβ expression in a systematic way^27^. Studying such drivers of variation allows researchers to build a greater understanding of how myriad distinct mutations converge to trigger the cascade of events that lead to symptomatic AD^12^. The above-mentioned study directions are just some of the current and future work of the seven major cores that together make up the DIAN collaboration. Like the neuroimaging data described within, much of these cross-modal data are available by request.

Despite the clear utility of the DIAN-OBS dataset, it is important to acknowledge that data heterogeneity is an inherent challenge for all international, longitudinal, neuroimaging studies^50^. Two main factors contribute to this variance: the availability of scanner hardware across sites and changes to data acquisition protocols that arise as improvements in technology are made over time. To minimize the influence of these factors, the DIAN Imaging Core has implemented several important measures. First, all image acquisition protocols are centrally designed in line with established AD imaging protocols of the ADNI study. These protocols are carefully tested before being deployed, and all sites are trained on the correct implementation of protocols. Prospective sites go through strict quality control checks, including scanning of a study-wide phantom to ensure that protocols are accurately run. After acquisition, all images are checked slice by slice to ensure that no protocol deviations or major movement artifacts are present, and, when necessary, sites may be asked to repeat a scan. Finally, images are run through standardized pre-processing pipelines, which, together with the strict quality control regime, result in imaging data with remarkably little variance. Nevertheless, potential residual effects may exist in the imaging data, and it may be of interest to some researchers to consider applying statistical harmonization procedures^51^. Notably, the DIAN Imaging Core provides both pre-processed and original source image data, along with scanner model information, allowing researchers to individually decide what method, if any, to apply to the data for any given analysis.

A major aim of the DIAN-OBS was to create an extensive data resource to be shared with a wide variety of researchers around the world. To that end, the data outlined in this paper are freely available by request and are organized in a manner that preserves participant privacy when shared. Although the DIAN-OBS is directly of interest to those studying ADAD, the common pathophysiology underlying multiple forms of AD make this resource highly relevant to a much wider AD network. Additionally, the collation of data from a large cohort of healthy controls further expands the broad utility of this data resource. The extensively phenotyped individuals within this dataset represent a relatively diverse cohort, with data acquired from over 21 sites across the globe. At the point of this writing, data release 15 is available, with additional data releases planned biannually. Given the rich data that the DIAN-OBS has collated and is continuing to generate, the authors hope that this resource paper will help to outline the neuroimaging data that can be requested.

## Methods

The imaging protocols for the DIAN-OBS contain complementary acquisitions chosen to represent the most sensitive measures for detecting and understanding preclinical AD-related pathology^52,53^. During the planning phase, considerations were made to accommodate concerns around time constraints, generalizability and data harmonization. A major strategic decision was made to not require participants to know their mutation status. Therefore, throughout the course of the DIAN-OBS, it has remained of paramount importance to not inadvertently reveal mutation status to participants who have not chosen to know this information. Given that a major aim of the DIAN-OBS was to create an open scientific resource, the source data and resulting pre-processed data are available by request (https://dian.wustl.edu/our-research/observational-study/dian-observational-study-investigator-resources/). The main goal of this paper is to provide a clear guide for the use of the DIAN-OBS imaging data as well as to supply researchers with information regarding data acquisition, pre-processing and relevant technical considerations. Data described represent the DIAN-OBS data release 15, encompassing data collected from February 2008 through December 2020. No statistical methods were used to predetermine sample sizes for any of the tests reported within this resource. Sample sizes for all figures, tables and analyses were determined by the availability of data such that the maximum number of data points were included. Discrepancies across the modalities reflect differences in completion of the various scan types or failures of specific data points to meet quality control standards. Moving forward, the DIAN-OBS data release will be updated with new data biannually.

### DIAN sites

The DIAN-OBS was launched in 2008 with 10 sites. Since its inception, this study has grown to include a total of 21 centers that span the Americas, Australia, Asia and Europe (Fig. 5). PET and MRI scanners at all sites are required to meet minimum hardware specifications to maximize the uniformity of collected data and ensure equivalent image quality across sites. All MRI scans were acquired on a 3T machine, and PET scans were acquired on one of the following PET scanner models: Siemens HR+, Siemens Biograph TruePoint PET/CT, Siemens Biograph mCT PET/CT, Siemens Biograph mMR, Siemens Biograph Vision PET/CT, Siemens High Resolution Research Tomograph (HRRT), Siemens Biograph 1023/1024, GE Discovery PET/CT or Phillips PET scanner. Before any participants being recruited at each site, acquisition accuracy was tested by running a traveling phantom through the imaging protocol. Subsequently, volunteer MRI scans were submitted to the Mayo Clinic, and volunteer PET scans were submitted to the University of Michigan teams for review to ensure that each site’s hardware was able to produce images of sufficient quality in line with common ADNI protocols^52,53^.

### Participants

Data from 534 participants, across 205 families with 108 different ADAD mutations spanning the *PSEN1, PSEN2* and *APP* genes, are included in the most recent DIAN-OBS data release (data release 15). These participants were recruited through DIAN collaboration sites, local initiatives at these sites and broader efforts (for example, http://dian-info.org/, http://www.alzforum.org/new/detail.asp?id=1967, http://www.alz.org/trialmatch and http://www.dianexpandedregistry.org/). The DIAN-OBS recruits second-generation members of families with known ADAD mutations, resulting in participants having a 50% chance of inheriting the mutation that exists within their family. An individual’s mutation status is determined by genotyping, but is not automatically revealed to the participant. Independent genetic counseling and testing are made available to all participants. Mutation non-carriers are used as well-matched study controls for mutation carriers. Participants were assessed on a battery of cognitive and clinical assessments every 3 years, unless the participant showed cognitive symptoms or was within 3 years of their EYO, in which case these tests were performed annually (Fig. 6).

All participants provided informed consent to be included in the ongoing DIAN-OBS. Furthermore, all study procedures were approved by the Washington University Human Research Protection Office, which serves as the central institutional review board (IRB). Local IRBs of the participating sites also approved all study procedures.

#### Clinical ratings.

Participants were assessed using CDR scales to determine their dementia status^18^. An overall CDR score was determined by evaluating ratings in memory, orientation, judgment, problem solving, function in community affairs, home and hobbies and personal care. Using the resulting scores, individuals were classified as cognitively unimpaired (CDR = 0) or having very mild (CDR = 0.5), mild (CDR = 1) or moderate-severe impairment (CDR > 1). In addition to CDR, participants were given a primary diagnosis as to the cause of any impairment.

EYO was assessed at each visit. EYO was calculated based on the participant’s current age, relative to their ‘mutation-specific’ estimated age of dementia onset, and also took into account the age that their parent became symptomatic^5^. The mutation-specific expected age of dementia onset was computed by averaging the reported age of dementia onset across individuals with the same mutation type. If the mutation-specific estimated age at dementia onset was unknown, the EYO was calculated from the age at which parental cognitive decline began. The parental age of clinical symptom onset was determined by a semi-structured interview with the use of all available historical data provided by the participant or their caregiver. The EYO was calculated identically for both mutation carriers and non-carriers and updated for mutation carriers upon their symptomatic conversion. All study staff performing clinical assessments were blinded to a participant’s mutation status.

### Protocol design and rationale

The DIAN-OBS neuroimaging protocol was designed by members of the DIAN Imaging Committee, in consultation with AD imaging experts, to closely align with the imaging protocols of ADNI^52,53^. This decision allowed the DIAN-OBS Imaging Core to leverage imaging expertise gained by ADNI regarding the successful deployment of longitudinal imaging protocols across multiple study sites. Aligning with the ADNI protocol also makes the DIAN-OBS imaging data accessible for researchers wanting to compare outcomes associated with ADAD to sporadic AD. Additionally, the DIAN Imaging Committee was responsible for outlining the process of onboarding study sites, which scanner models would be accepted, quality control procedures and data pre-processing pipelines. Major considerations included ensuring that imaging data were collected in a manner that maximized data utility and participant compliance while also minimizing participant burden. For example, the acquisition of T1w images were acquired early in the MRI session, when movement is least prevalent, as they are critical for the pre-processing of several other MRI-acquired and PET-acquired images. Given the longitudinal nature of the DIAN-OBS, the DIAN Imaging Committee also convenes as needed to discuss potential changes in imaging protocols.

The first iteration of the DIAN-OBS imaging protocol (DIAN-1) included three complementary imaging acquisitions aiming to quantify Aβ accumulation (PiB-PET), glucose metabolism (FDG-PET) as well as structural and functional dysfunction (MRI). Across the course of the DIAN-OBS, the imaging protocol has undergone two major shifts (DIAN-2 and DIAN-3). These shifts reflect advances in scanner technology, tracer development and changes to ADNI imaging protocols. Specifically, the implementation of the DIAN-2 protocol in 2012 marked the introduction of arterial spin labeling (ASL), an extension to the MRI protocol of DIAN-1. Similarly, the subsequent introduction of the DIAN-3 protocol in 2018 marked a shift from two-dimensional (2D) to three-dimensional (3D) ASL, upgraded the diffusion-weighted sequence to be compatible with diffusion basis spectrum imaging (DBSI) and introduced tau-PET. Notably, these changes were made after careful discussion between members of the DIAN-OBS Imaging Committee and outside field experts.

### Image acquisition: PET

When the DIAN-OBS was first initiated, PET scans to measure Aβ deposition and brain glucose metabolism were performed using a modified version of the ADNI PET acquisition protocol^53^. However, to support developing interest in understanding the role of tau accumulation, recent changes in the DIAN imaging protocol have been made to integrate tau-PET scanning. Detailed below are the acquisition parameters of each of these three distinct PET imaging modalities.

#### PiB-PET.

To quantify the spatial patterns and magnitude of Aβ deposition, PiB-PET imaging was completed using a single bolus injection of approximately 15 mCi of [^11^C]PiB. PiB-PET scans were subsequently collected for either 70 min immediately after injection or across a 30-min time window that began after a 40-min post-injection delay. For analyses, only the common 30-min period for each scan variant was used. Example PiB-PET images are displayed for each of the three participant group types in Fig. 6.

#### FDG-PET.

To measure the spatial patterns and rate of glucose metabolism, approximately 5mCi of [^18^F]FDG was given via a single bolus injection. Once the tracer was administered, a delay of 30 min was observed before the PET emission data were acquired for a period of 30 min. For analyses, the last 20-min period of each scan was used. Example FDG-PET images are displayed for each of the three participant group types in Fig. 3.

#### Tau-PET.

Given that the accumulation of tau has also been described as a characteristic pathology of AD^14,37^, tau-PET imaging is currently being added to the DIAN imaging protocol. To accommodate varying availability of tau-validated tracers across the globe, three tau-PET tracers are currently being added to the DIAN-OBS: [^18^F]MK-6240, [^18^F] AV-1451 (flortaucipir) and [^18^F]PI-2620. At this writing, two of these tracers are actively being used by DIAN-OBS sites for data collection: [^18^F]MK-6240 and [^18^F]AV-1451, with most of the data being acquired with [^18^F]AV-1451. For [^18^F]MK-6240, a single bolus injection of 5 mCi of this tracer is given to participants, with images dynamically acquired for 110 min after injection, whereas, for [^18^F]AV-1451, a single 10-mCi bolus injection of this tracer is administered to participants, and images are acquired dynamically for the next 105 min. SUVRs are calculated over the 80–100-min and 90–110-min post-injection windows for [^18^F]AV-1451 and [^18^F]MK-6240, respectively. Example tau-PET images for each of these two tracers are displayed in Fig. 3.

### Image acquisition: MRI

Several MRI modalities were employed to visualize brain structure and function within the DIAN cohort. The order of scan collection was optimized to maximize participant compliance. All scans were collected on a 3T scanner using parameters described below, although scanner model and manufacturer vary by site.

#### T1w structural scan.

High-quality T1w scans providing information relating to brain volume and morphology are integrated into the pre-processing and registration of other collected scans (for example, resting state MRI (rsMRI)) and are critical to the anatomical registration of PET data. The T1w magnetized prepared rapidly acquired gradient echo (MPRAGE) sequence in DIAN-OBS was matched to the ADNI MRI protocol^52^, with the following parameters: echo time (TE) = 2.95 ms, repetition time (TR) = 2,300 ms, inversion time (TI) = 900 ms, field of view (FOV) = 270 mm, flip angle = 9°, number of slices = 225, voxel size = 1.1 × 1.1 × 1.2 mm^3^, GRAPPA acceleration factor = 2 and acquisition time = 5 min, 12 s. Example T1w images are displayed for each of the three participant group types in Fig. 3. To date, studies have used information derived from these high-quality T1w images to determine structural patterns unique to ADAD^26,36,54,55^.

#### Diffusion-weighted images.

Diffusion-weighted scans are specifically sensitive to the thermal motion of water over time and, therefore, can be used to infer the presence of biological structures within the brain. Given the highly uniform nature of white matter within the brain, water movement tends to be especially constrained where axons are present^56^. This property is not maintained within the CSF or gray matter structures, where water tends to diffuse in a much less constrained manner given that there are relatively fewer biological boundaries in these structures^57^. These properties of water movement make diffusion-weighted images (DWIs) uniquely positioned to measure the integrity of white matter microstructures within the brain. To this end, we initially acquired diffusion-weighted scans with the following parameters: TE = 81 ms, TR = 7,000 ms, FOV = 256 mm, number of slices = 60, voxel size = 2 × 2 × 2 mm^3^, maximum diffusion weighting = 2,000 s mm^−2^ and acquisition time range = 2 min, 43 s to 3 min, 4 s. These traditional diffusion tensor-optimized scans were phased out in 2017 in favor of optimizing DWIs for DBSI in the DIAN-3 MRI protocol.

The DIAN-DBSI sequence comprises three diffusion sequence sessions with the Siemens built-in 6, 10 and 12 diffusion vectors, respectively. Multiple *b* values were implemented in each session. The maximal *b* values for each session are 2,000, 1,500 and 1,000s mm^−2^, respectively. By combining all three sessions, 28 unique directions were acquired, with 66 unique diffusion weightings. For each run, there was one volume with no diffusion weighting (*b* = 0 s mm^−2^) accounting for the remaining volumes. Together, the acquisition time was 9 min, 14 s. This unique sequence design allows for DBSI algorithms to subsequently implement neuroinflammation imaging, an important branch of diffusion research in preclinical AD^58^. Example DWIs are displayed for each of the three participant group types in Fig. 3.

#### T2-fluid-attenuated inversion recovery.

T2-fluid-attenuated inversion recovery (FLAIR) images are useful tools for identifying white matter hyperintensities, a phenomenon known to be increased in individuals with sporadic and autosomal dominant AD^39,59^. These scans complement DWIs by providing information on the integrity of white mater macrostructure. Based on the ADNI MRI protocol, axial T2-FLAIR images were acquired with the following parameters: TE = 91 ms, TR = 9,000 ms, TI = 2,500 ms, FOV = 220 mm, flip angle = 150°, slices = 35, voxel size = 0.9 × 0.9 × 5 mm, acceleration factor = 2 and acquisition time =4 min, 5 s. Example T2-FLAIR images are displayed for each of the three participant group types in Fig. 3.

#### Gradient recalled echo-based sequences.

T2-Star gradient recalled echo (GRE) sequences, as well as susceptibility-weighted images (SWIs), can be used to characterize pathological changes occurring to venous vasculature within the brain. More specifically, GRE sequences are sensitive to hemorrhage, calcification and iron deposition^60^, allowing researchers to detect the presence and location of cerebral microbleeds, a common pathology associated with ADAD^38,40,61,62^. Following the ADNI MRI protocol, axial T2-Star scans were acquired with the following parameters: TE = 20 ms, TR = 650 ms, FOV = 200 mm, flip angle = 20°, slices = 44, voxel size = 0.8 × 0.8 × 4 mm^3^ and acquisition time = 4 min, 11 s. Example T2-Star images are displayed for each of the three participant group types in Fig. 3. SWIs (TE = 20 ms, TR = 28 ms, flip angle = 15°, slices = 88 and voxel size = 0.7 × 0.7 × 2 mm^3^) were also originally acquired at sites that could not collect T2-Star images due to scanner limitations. To harmonize across site and vendors, all sites are now acquiring T2-Star GRE.

#### 2D or 3D pulsed ASL.

ASL is an MRI technique that can measure blood perfusion without the use of an exogenous contrast agent and can be used to assess qualitative or quantitative cerebral blood flow. Previous studies using ASL have reported hypoperfusion in the posterior cingulate, precuneus and parietal cortices in individuals with AD compared to healthy controls^63-65^. However, few studies using ASL have focused on ADAD, and further investigations in this population are needed^66,67^.

The multi-site and international nature of DIAN necessitates use of readily available, standardized and vendor-provided ASL protocols, so protocols were harmonized to ADNI (https://adni.loni.usc.edu/). Example images are displayed in Fig. 3, and sequence parameters are available in Extended Data Table 2.

#### Functional MRI.

rsMRI scans measure fluctuations in the blood-oxygen-level-dependent (BOLD) signal. Correlations in these spontaneous BOLD fluctuations are thought to reflect intrinsic functional connectivity within and between brain networks^68^. Previous work has shown that individuals with AD exhibit abnormal patterns in rsMRI connectivity, particularly in the default mode network^69^. Given that this network comprises regions already implicated in AD pathology^70^, rsMRI is a promising tool for investigating network disruption caused by ADAD progression. The following parameters were used to acquire a subset (*n* = 394) of rsMRI scans at sites employing Siemens scanners: TE = 30 ms, TR = 2,230 ms, flip angle = 80°, acquisition matrix = 64 × 58 × 36 and voxel size = 3.3 × 3.3 × 3.3 mm. Example default mode network images derived from rsMRI data using these parameters are displayed for each of the three participant group types in Fig. 3. Additional rsMRI scans were collected using a variety of scanner models and imaging parameters. A summary of rsMRI sequence parameters is provided in Extended Data Table 3. All rsMRI scans were acquired with single-band protocols for 5.13 min while participants rested with their eyes open.

#### T2-fast spin echo.

In addition to the above scans, a T2-fast spin echo (FSE) scan was also acquired as part of the MRI protocol. The main purpose of this scan is to assist in registration efforts for the rsMRI scans collected. T2-FSE scans were collected with the following parameters: TE = 563 ms, TR = 3,200 ms, FOV = 270 mm, slices = 225, voxel size = 1 × 1 × 1.2 mm, GRAPPA acceleration factor = 2 and acquisition time =4 min, 8 s. Example T2 FSE images are displayed for each of the three participant group types in Fig. 3.

### Quality control and data quantification

Once acquired, raw data were transferred from the DIAN-OBS scanners to sites of quality control assessments via a standardized protocol. MRI images were assessed by experts of the ADNI Imaging Core at the Mayo Clinic, and PET scans were assessed by a team of experts at the University of Michigan. Each of these sites is responsible for the support, management and primary quality control of their relevant imaging modality (Extended Data Fig. 4). All incoming imaging files were assessed for protocol compliance, clinically meaningful medical abnormalities and image quality, using a combination of automated and manual processes. Once initial quality control has been passed, MR and PET data are stored in the DIAN Central Archive, an XNAT-based archive. Image processing occurs in the DIAN Imaging Core at Washington University, and post-processed data for the data releases are provided to the DIAN Biostatistics Core for incorporation into formal data releases.

#### MRI processing.

T1w images were pre-processed using the FreeSurfer software suite (version 5.3-HCP-patch, http://surfer.nmr.mgh.harvard.edu/)^25^. Structural images were corrected for motion artifacts, and then a hybrid watershed and surface deformation procedure was used to remove the brain from the skull. Images were then registered to Talairach space for subcortical white matter and gray matter structures to be segmented. A tessellation step was then employed to estimate the gray and white matter structural boundary and apply any necessary topological correction. All intersurface boundaries were placed in their optimal locations using surface normalization and intensity gradients. Finally, images underwent surface inflation and registration to a spherical atlas.

#### PET processing.

PET data were analyzed using the PET Unified Pipeline (PUP, https://github.com/ysu001/PUP)^24^. PUP includes scanner resolution harmonization to a common full width at half maximum, inter-frame motion correction using the summed image as the reference, PET to MRI registration, extraction of time activity curves for each FreeSurfer-defined region of interest from the Desikan atlas, SUVR computations for each region of interest and a partial volume correction procedure adjusting for regional spill-in and spill-out using a calculated regional spread function implemented in a geometric transfer matrix approach. If dynamically acquired PET data are available (*n* = 324), PUP will additionally calculate non-displaceable binding potentials (BPs) using a Logan graphical analysis method. Quantitative modeling was performed on the post-injection time windows of 40–70 min and 40–60 min for PiB and FDG, respectively, with cerebellar gray chosen as the reference region.

In cases where a matched MPRAGE fails quality control checks, image processors will initiate a secondary pipeline to manually segment PET images, allowing PET processing to continue. Whenever manual segmentation is required to process PET data, the image processing technicians will also process all within-subject longitudinal PET visits using manual segmentations to ensure consistency across visits. It is not recommended to use both manual and FreeSurfer-derived PET data within a single analysis, but, where this is unavoidable, it is best practice to use FreeSurfer values that have not been corrected for partial volume effects.

#### Post-processing quality checks.

Scientists of the DIAN Imaging Core at Washington University manually inspected all output of the FreeSurfer and PUP processing. Any images requiring manual intervention were corrected, and processing was rerun to ensure consistency across scans. There are two main types of FreeSurfer errors: inclusion and exclusion. An inclusion error occurs when non-brain regions are identified by FreeSurfer as brain matter, whereas exclusion errors occur when brain regions are incorrectly ignored by FreeSurfer segmentation. Up to three attempts were made to fix FreeSurfer errors that persisted after intervention. If errors continued to persist after the third attempt, the FreeSurfer was considered to have failed quality control, and data were quarantined from release.

Although many DIAN-OBS images required no manual edits, common errors did occur in the data that require these interventions. For example, atrophy of the ventricles, degradation of white matter structures and regions of low signal greatly reduced the accuracy of FreeSurfer segmentations. Although in most cases edits rectified these issues, they did preferentially impact estimates of regions important to AD research, the ventricles, hippocampus and amygdala. Increased pathology and motion were also associated with FreeSurfer failure rate. Notably, these underlying drivers of FreeSurfer errors are likely to be disproportionately present in symptomatic individuals. For this reason, much effort has been undertaken to inspect and correct these errors, to ensure that most DIAN-OBS scans are retained in the overall dataset, with the least possible edits. A summary of DIAN-OBS edits and failures are provided in Extended Data Fig. 5.

### Processed image calculations

In addition to making source data available, the image processing pipeline results in output that represents structural volume and thickness as well as quantification of PET tracer uptake. These measures are released by region, which are derived via the FreeSurfer segmentation applied during processing. In addition to regional values, several global summary variables are also available within the DIAN-OBS data release. For MRI releases, thesse data represent volume or thickness, whereas PET data represent mean BP or SUVR. Below, we define global summary measures and provide additional context for proper use of DIAN-OBS imaging variables.

#### Summary cortical Aβ.

The DIAN-OBS imaging release provides a summary cortical Aβ measure, based on the arithmetic mean of the SUVR (or BP) from the precuneus, prefrontal cortex, gyrus rectus and lateral temporal regions. This summary measure has been previously defined and validated as a sensitive measure of Aβ in individuals with preclinical AD^24^.

#### Summary cortical thickness.

This variable provides researchers with a summary cortical thickness measure that was developed to capture cortical atrophy that is specific to ADAD pathology and has been previously validated^26^. This summary metric was created using vertex-wise analyses to determine a mask capturing the cortical regions that most closely associated with change in individuals with this disease. Roughly, this measure captures cortical atrophy across left isthmus cingulate, the left and right precuneus and right hemisphere inferior parietal, superior parietal and lateral occipital regions (see Dincer et al.^26^ for full details).

#### Partial volume correction.

As PET images have low spatial resolution, measured signals are distorted by partial volume effects. The extent that these effects influence PET output is dependent upon the size and shape of the region of interest as well as the spatial resolution of the scan. To account for the distortions that these effects introduce, a common correction technique based on regional spread function is implemented in our processing pipeline^71^. Prior work has confirmed that using this technique was able to improve PET quantification and sensitivity to detecting Aβ burden^72^. In the DIAN-OBS imaging data release, both uncorrected and corrected data are available, to allow researchers to independently judge whether to employ this correction for their specific analyses.

#### Centiloid conversion.

The DIAN-OBS does not release Aβ-PET data in centiloid units. Centiloid units allow researchers to compare data collected across a variety of Aβ-PET tracers and acquisition parameters by converting mean cortical SUVR (or BP) into a measure of global Aβ deposition. Notably, the reference region chosen for PET analyses greatly influences the estimation of mean centiloid values. Although PiB-PET centiloid equations have been validated using the cerebellar cortex, whole cerebellum and brainstem as reference regions, cerebellar cortex is thought to have lowest variability in younger individuals^73^. The DIAN-OBS processing pipeline uses the cerebellar gray matter as the reference region, and prior work has illustrated that implementing standard centiloid analyses on this data yields output that strongly corresponds with published centiloid measures^74^. Taken together, the released DIAN-OBS is suitable for conversion to centiloid units using relevant equations (Extended Data Table 4), although this is currently validated only for our summary measure.

#### Volumetric normalization.

Finally, it is strongly recommended that MRI regional volumes are corrected for an individual’s intracranial volume to ensure that valid inferences can be made from comparisons across participants or groups. To perform this correction, the following calculation should be made:

Normalizedvolume=regionalVol−(B−weight∗(ilCV−sampleICV))

Where the *B – weight* is derived from a regression modeling the relationship between a specific regional volume (*regionalVol*) and an individual’s intracranial volume (*iICV*), and *mICV* is the average ICV for the study sample. This correction must be applied separately for each specific FreeSurfer region, given that head size differentially impacts volume in a regional manner^75^. Notably, this normalization to ICV is not necessary for regional measures of cortical thickness, as this does not substantially vary with head size.

## Extended Data

**Extended Data Fig. 1 ∣:**
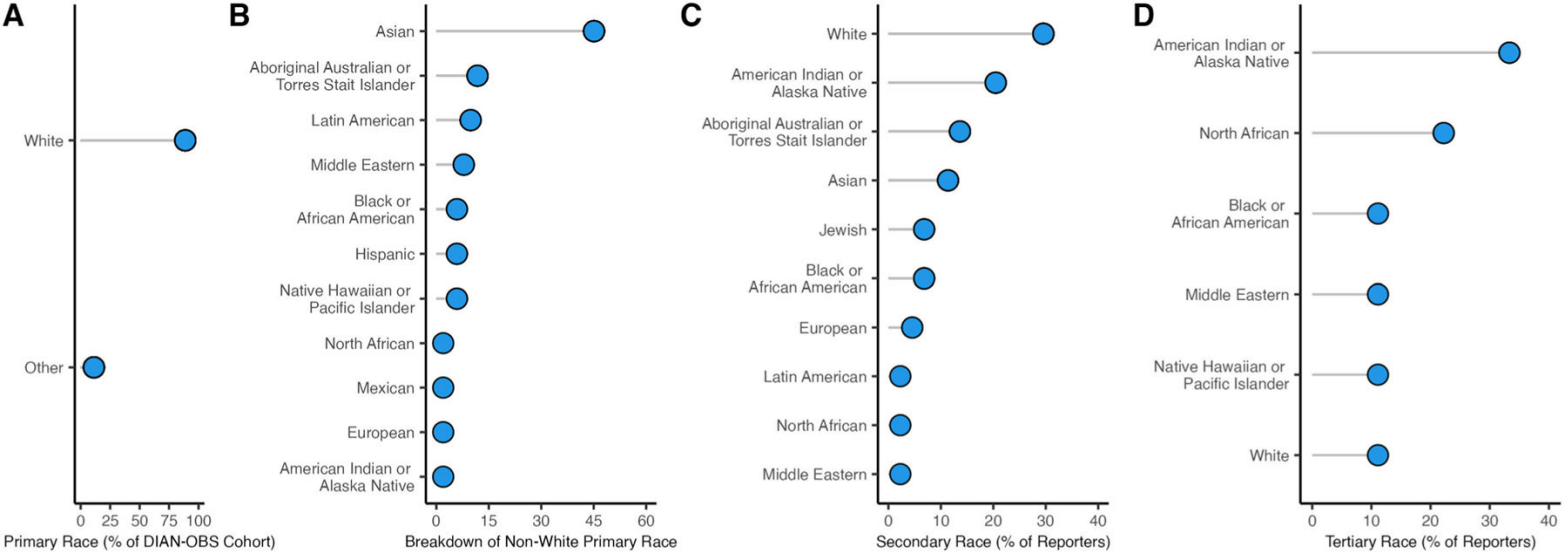
Extended breakdown of self-reported race of participants in the DIAN-OBS. **A:** The majority of individuals In DIAN-OBS, self-report their primary race as white (*n* = 474). For visualization all other self-reported race outcomes were grouped as ‘other’ (*n* = 60), and are visualized in plot B. **B:** Depiction of the breakdown of non-white primary self-reported race (*n* = 60). **C:** Visualizations of the 44 individuals from the DIAN-OBS also reported a secondary self-identified race affiliation, and, **D:** 9 individuals reported a tertiary self-identified race affiliation.

**Extended Data Fig. 2 ∣:**
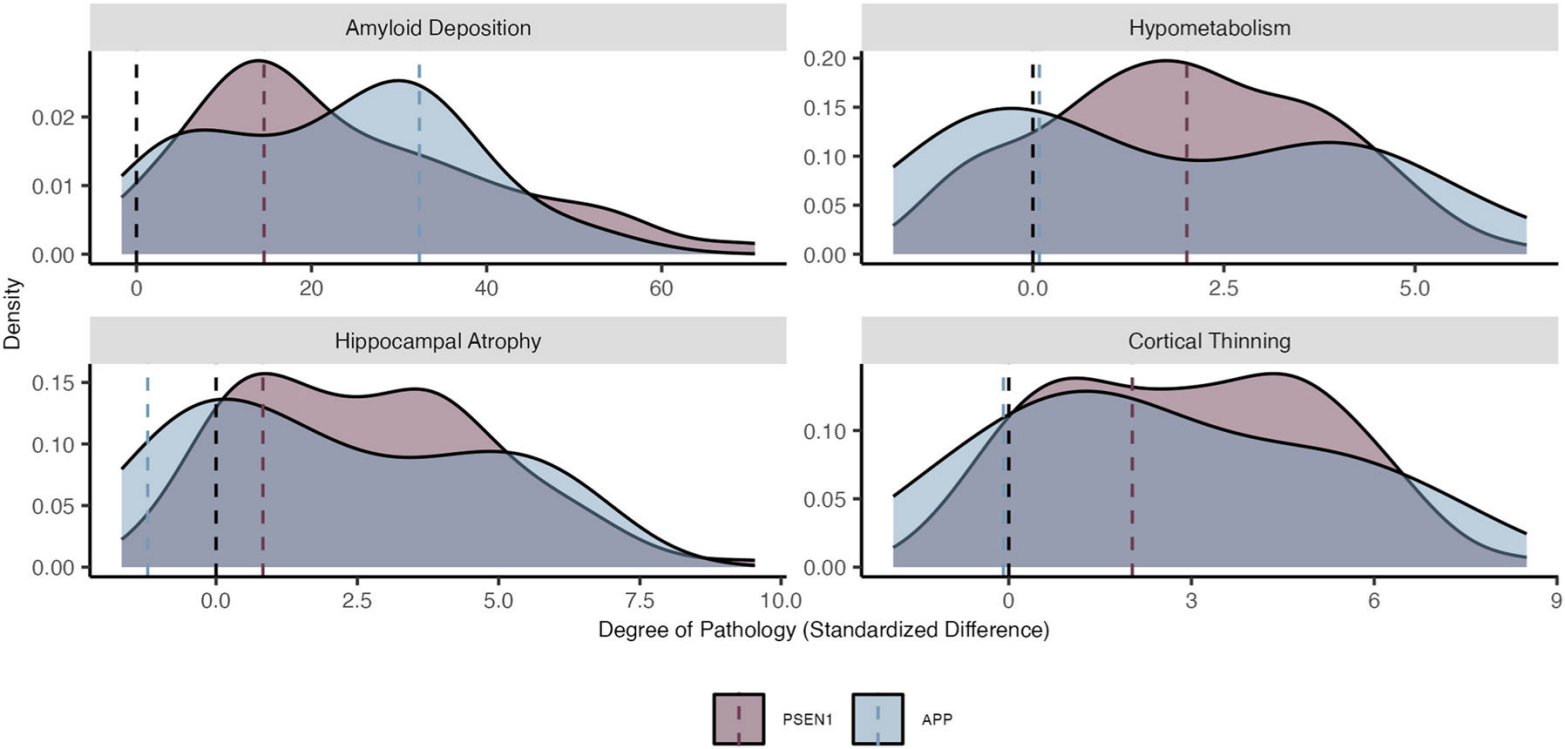
Phenotypic heterogeneity for key imaging outcomes by ADAD mutation type. A density plot representing the distribution of pathological accumulation of common biomarkers of ADAD. Here, separate density curves are plotted for carriers of the *PSEN1* (purple) and *APP* (blue) mutations. While we typically think of ADAD as a homogenous form of AD, each mutation conveys a variable impact on the phenotypic expression of these common biomarkers. Values represent z-scores relative to the unimpaired mutation non-carriers. The black dashed line represents the mean value for mutation non-carriers, while colored dashed lines represent mean z-scores for each group, respectively. Amyloid deposition represents PiB SUVR uptake (*n* = 281), hypometabolism is derived from FDG SUVR update (*n* = 296), cortical atrophy is a measure of cortical thickness (*n* = 318), hippocampal atrophy is a measure of hippocampal volume (*n* = 318), clinical symptoms represent MMSE scores (*n* = 316), and cognitive decline represents accuracy on a composite of general cognitive tasks (*n* = 305). *Plot demographics: n = 318, average age = 39.4, proportion females = 56%*.

**Extended Data Fig. 3 ∣:**
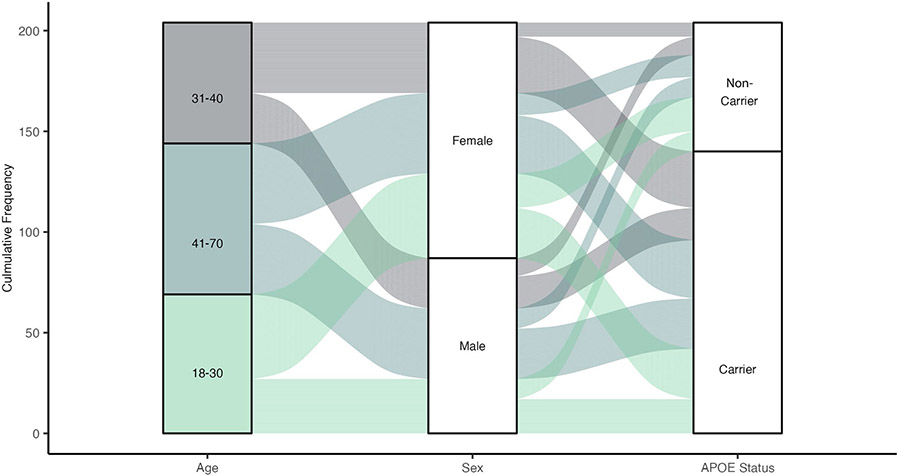
Visual depiction of the demographic characteristics of the control participants in the DIAN-OBS. Of the 534 participants in the DIAN-OBS, 204 are unimpaired non-carriers of the ADAD mutations. Here, we visualize the proportions of these 204 individuals who fall into each age bracket, showing that 2/3 of these individuals are under the age of 40. This depiction also highlights the relative proportions of unimpaired non-carriers that are male and female, ast well as the proportions of these individuals who are carriers of at least one copy of the *APOE ε4* allele. *Plot demographics: n = 216, average age = 37.1, proportion females = 57%*.

**Extended Data Fig. 4 ∣:**
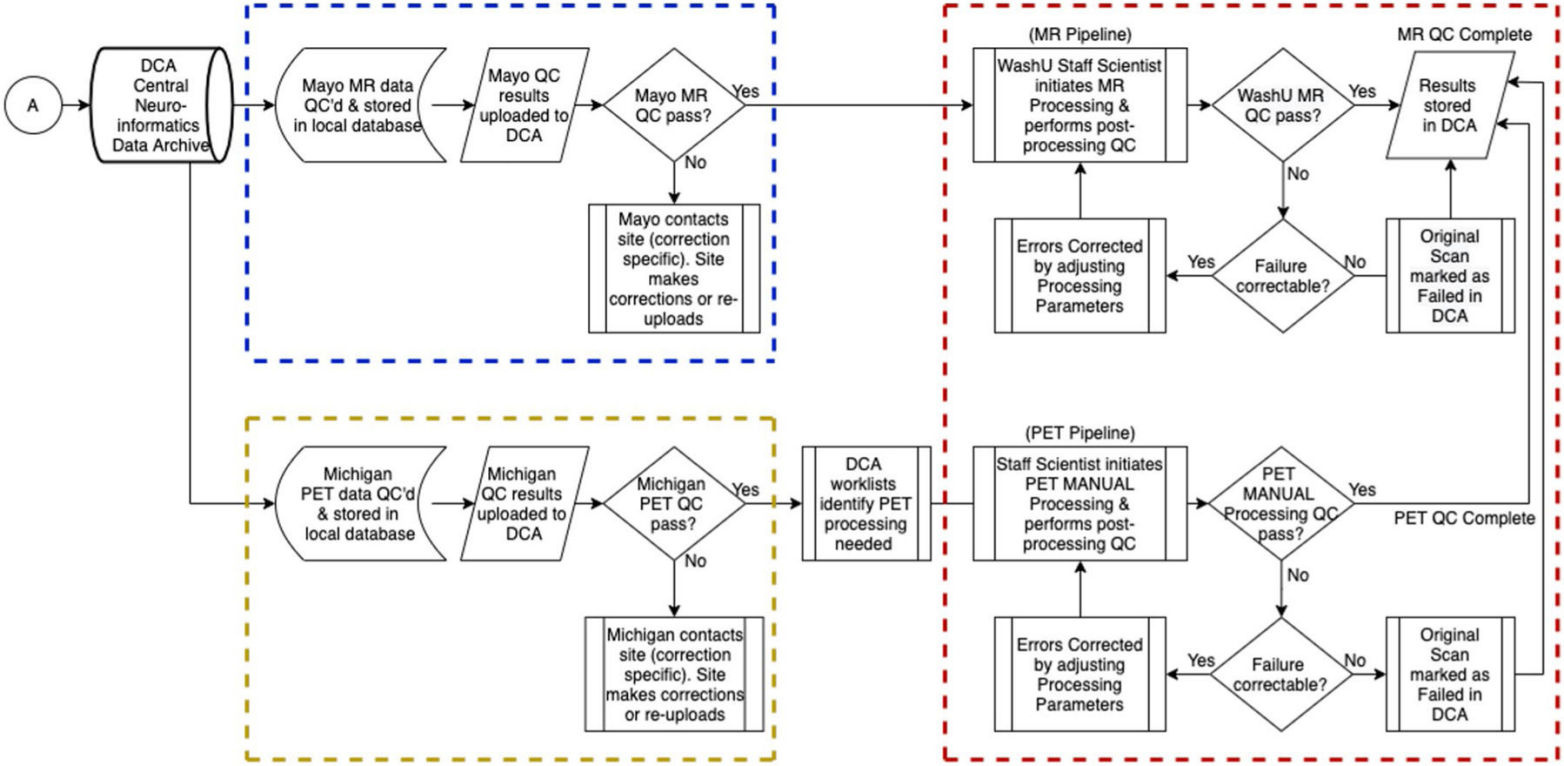
Flow chart depicting the quality control workflow for the DIAN-OBS. Mayo Clinic, Rochester, MN, is responsible for the support, management, and primary quality control procedures for MRI, and for the participant safety reads (dashed blue lines). The University of Michigan, Ann Arbor, MI, is responsible for the support, management, and primary quality control of PET participant sessions (dashed yellow lines). Once initial quality control has been passed, the MR and PET data are stored and processed in the DIAN Central Archive, an XNAT-based archive. Staff at Washington University School of Medicine are responsible for initial processing of MRI and PET images, and subsequently organizing data into each publicly accessible data release (dashed red lines).

**Extended Data Fig. 5 ∣:**
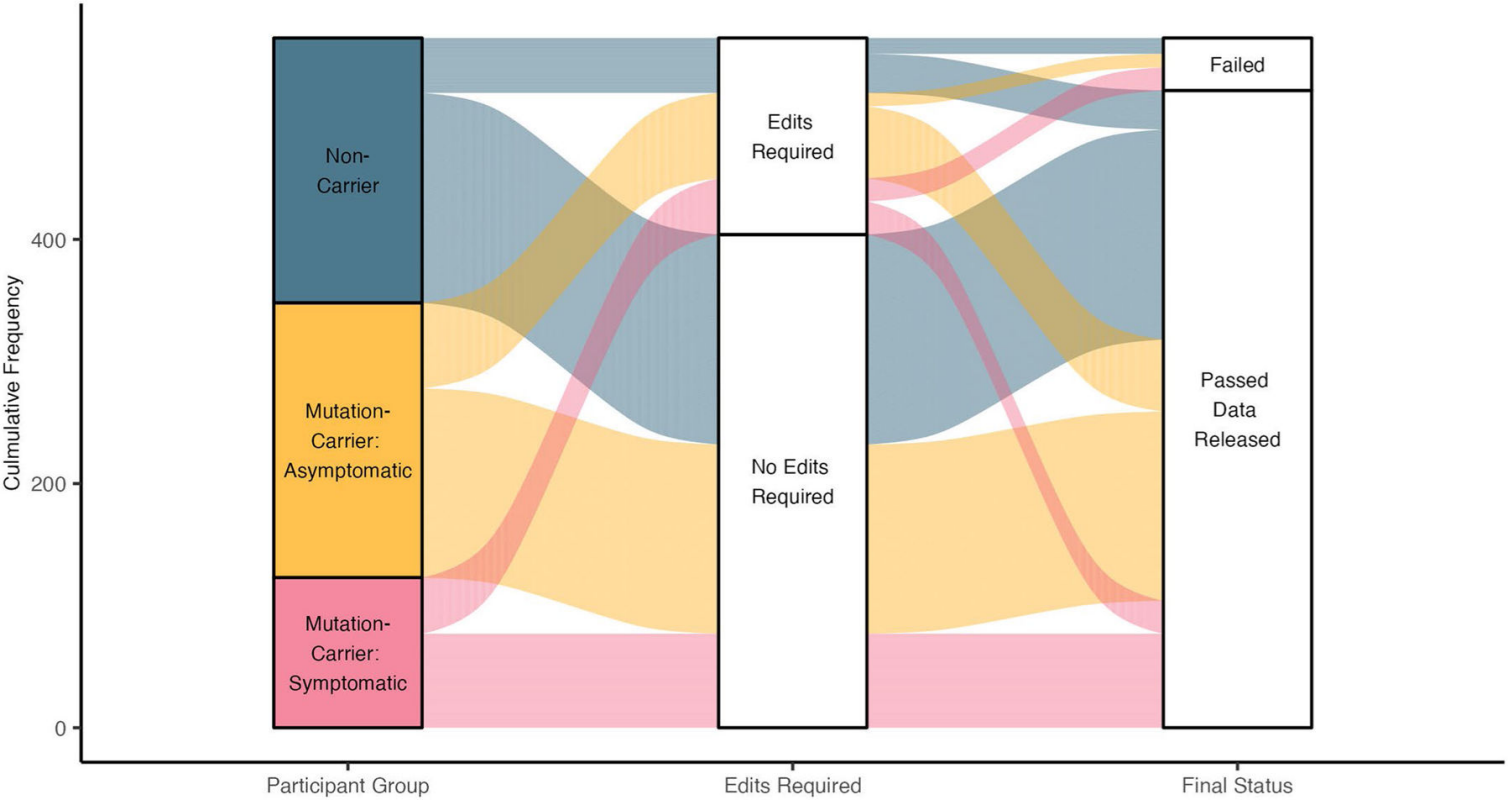
Visual flow chart showing the proportions of T1w images that require editing during the quality control process. Here we show the relative pass-rate of T1w images undergoing FreeSurfer processing at Washington University. Column one splits the images by impairment and mutation carrying status, while column two shows the relative number of images that pass the initial round of FreeSurfer processing without intervention (72%), and column three indicates the final proportion of images that are able to be included into the DIAN-OBS data release (93%). Between column two and three, technicians of the DIAN-OBS Imaging Core carefully edit images to correct for minor FreeSurfer errors and allow the image to repeat FreeSurfer processing. Importantly, only a small proportion of DIAN-OBS images cannot be recovered through this editing process and will not be included in released data, depicted here as “Failed” (7%). This plot also illustrates that while a larger proportion of symptomatic mutation carriers do require edits, images that are withheld from the final data release represent all three participant groups.

**Extended Data Table 1 ∣:** Summary results of the PET and MRI comparisons for the three participant groups

		Mutation non -carriers	Mutation- carriers: asymptomatic	Mutation- carriers: symptomatic	*p*-value	*Effect* *size*
MRI	*n*	216	214	104	-	-
	Hippocampal volume (*SE*)	8848.97 (45.57)	8881.26 (*53.37*)	7440.18 (*132.84*)	*1.07 x 10^−29^*	0.30
	Cortical thickness** (*SE*)	2.34 (*0.01*)	2.33 (*0.01*)	2.07 (*0.02*)	*2.53 x 10^−1^*	0.36
PiB-PET	*n*	203	199	82	-	-
	Summary cortical PiB*** (*SE*)	1.06 (*0.01*)	1.57 (*0.05*)	2.71 (*0.13*)	*4.62 x 10^−51^*	0.43
FDG-PET	*n*	198	205	91	-	-
	Summary FDG**** (*SE*)	1.71 (*0.01*)	1.68 (*0.01*)	1.47 (*0.02*)	*5.34 x 10^−21^*	0.23

These analyses reveal that symptomatic mutation carriers show greater pathology than asymptomatic mutation carriers and non-carriers, as measured by atrophy, rates of glucose metabolism and Aβ accumulation. All depicted variables represent mean values with s.e. in parentheses. Statistical tests are one-way ANOVAs, and effect sizes represent partial η^2^.

**Extended Data Table 2 ∣:** Parameters of the ASL sequence employed in the DIAN-OBS

Vendor	Sequence	Tag	Resolution (mm)	TR (ms)	TE (ms)	TI1 (ms)	TI (ms)
Siemens	2D PASL	PICORE	3.2 x 3.2 x 4	3400	13	700	1900
Phillips	2D PASL	STAR	3.2 x 3.2 x 4	3400	13	700	1900
Siemens	3D PASL	FAIR	1.9 x 1.9 x 4.5	4000	21.8	800	2000

Note: PASL=pulsed Arterial Spin labelled; PICORE=Proximal Inversion with Control of Off-Resonance Effect;, STAR=Signal Targeting by Alternating Radiofrequency pulses; FAIR=Flow-sensitive Alternating Inversion Recovery.

ASL was first introduced to the DIAN-OBS imaging protocol in 2012 with the implementation of DIAN-2. In 2018, the introduction of DIAN-3 marked the transition of this sequence to acquire images using a 3D ASL acquisition.

**Extended Data Table 3 ∣:** Summary of the full rsMRI imaging parameters

Vender	TR (ms)	TE (ms)	Flip Angle	Resolution (mm)	Acquisition Matrix	Slices
Siemens	2200-3050	30	80	3.3 x 3.3 x 3.3 or 3.4 x 3.4 x 3.3	58 x 64, 64 x 58, or 64 x 64	36-48
GE	2925-3000	30	90	3.3 x 3.3 x 3.3 or 3.3 x 3.3 x 3.6	64 x 64	42-54
Phillips	2200-2000	30	80	3.3 x 3.3 x 3.3	64 x 64	34-48

Presented here are ranges of parameters for the rsMRI sequences employed across the DIAN-OBS sites, by scanner model. In all cases, scans were acquired in the axial orientation, with a multiband factor of 1, and participants were asked to keep their eyes open.

**Extended Data Table 4 ∣:** Conversion equations for converting PiB-PET values to centiloid

Aβ PET Measure	Partial Volume Corrected	Equation
PiB-BP	No	Centiloid = 122.5 x PiB – 6.2
	Yes	Centiloid = 52.0 x PiB – 4.2
PiB-SUVR	No	Centiloid = 102.8 x PiB – 112.2
	Yes	Centiloid = 40.7 x PiB – 42.9

The four equations provided allow researchers to convert extracted cortical summary PiB-PET values to centiloid units. These equations cover both SUVR-derived and BP-derived measures with and without partial volume correction.

## Supplementary Material

1

## Figures and Tables

**Table 1 ∣:** Baseline demographic information for the participants included in the DIAN-OBS data release 15 (*n* = 534)

		Mutation non-carriers	Mutation carriers: asymptomatic	Mutation carriers: symptomatic	*P* value	Effect size
Cohort	*n*	216	214	104	–	–
	Age in years (s.e.)	37.10 (0.75)	33.75 (0.61)	45.14 (0.97)	9.88 × 10^−19^	0.15
	Sex: *n* (%) female	123 (57%)	120 (56%)	58 (56%)	0.97	0.01
	Handedness: *n* (%) right	187 (87%)	188 (88%)	94 (90%)	0.62	0.04
	Education in years (s.e.)	14.77 (0.20)	14.78 (0.19)	13.41 (0.34)	5.8 × 10^−4^	0.03
	*n* (%) White	196 (91%)	188 (88%)	90 (87%)	0.46	0.05
Clinical	EYO (s.e.)	−10.49 (0.79)	−14.30 (0.61)	2.79 (0.49)	–	–
	CDR: *n* (%) unimpaired	204 (95%)	214 (100%)	0 (0%)	–	–
Genetics	*n* (%) *PSEN1*	–	150 (70%)	82 (79%)		
	*n (%) PSEN1* pre-codon 200**	–	53 (35%)	30 (37%)		
	*n* (%) *PSEN2*	–	24 (11%)	1 (1%)		
	*n* (%) *APP*	–	40 (19%)	21 (20%)		
	*n* (%) APOE-ε4*	66 (30%)	63 (29%)	27 (26%)	0.70	0.04
Cognition	MMSE (s.e.)	29.63 (0.45)	29.36 (0.32)	23.68 (0.95)	2.03 × 10^−10^	0.11
	General cognition*** (s.e.)	0.04 (0.06)	0.11 (0.08)	−1.01 (0.19)	8.65 × 10^−11^	0.12

All *P* values are relative to the highest-level model, with α = 0.05. For age, all follow-up pairwise comparisons are significant after Bonferroni adjustment for multiple comparisons, for years of education, MMSE and general cognition; only *P* values for contrasts including symptomatic mutation carriers remain significant after Bonferroni adjustment for multiple comparisons.

** Pre-codon 200 values represent the percentage of *PSEN1* mutation carriers with mutations occurring before the 200th codon of *PSEN1*.

*** General cognition depicts averaged *z*-scores across four cognitive tests, computed relative to unimpaired mutation non-carriers with EYO between −10 and 0. Here, participants are categorized into three groups representing ADAD mutation-carrying status and level of cognitive impairment. All depicted variables represent mean values with s.e. in parentheses or percentages. Statistical tests were used to compare distributions of these characteristics across these three groups and are one-way ANOVAs or χ^2^ tests, where appropriate. Effect sizes represent partial η^2^ or φ, as appropriate. More detailed information regarding self-identified race is reported in Extended Data Fig. 1.

**Table 2 ∣:** Longitudinal availability of images by scan type and total number of scans by group

Sequence	Scans by longitudinal visit								Baseline scans available by group		
	1	2	3	4	5	6	7	8	Mutation non-carriers	Mutation carriers: asymptomatic	Mutation carriers: symptomatic
T1w	534	342	169	79	35	11	3	2	216	214	104
T2-Star	421	224	102	34	3	1	–	–	104	112	42
T2-SWI	284	88	18	3	–	–	–	–	116	97	61
ASL	392	185	87	21	1	1	–	–	101	101	32
DWI	496	285	136	66	24	10	3	1	180	170	70
T2-FLAIR	533	340	168	78	35	11	3	2	215	214	102
rsMRI	529	332	160	75	34	11	3	2	211	213	103
T2-FSE	402	203	87	28	2	1	–	–	93	99	36
PiB-PET	502	279	121	55	22	9	3	2	207	204	91
FDG-PET	509	281	123	54	26	7	3	–	204	210	95
Tau-PET (MK)*	27	4	–	–	–	–	–	–	13	13	1
Tau-PET (AV)**	50	20	8	2	–	–	–	–	23	23	4

Given global disparities in the availability of tau-PET tracers, two distinct compounds have been used across the DIAN-OBS:

* MK = MK-6240 and

** AV = AV-1451 Here, counts represent the number of images that have passed extensive quality control checks and can be requested as part of the DIAN-OBS data release 15.

## Data Availability

All data described in the current resource manuscript are freely available upon completion of a DIAN-OBS Data Request Form. This request procedure allows DIAN to implement a transparent and inclusive data-sharing practice while maximizing confidentiality and security of our participants’ protected personal information. Every submission is reviewed by the DIAN principal investigator as well as relevant DIAN core leaders (that is, the Imaging Core leader). Data requests are approved based on scientific merit and feasibility, appropriateness of the research goals and the possession of adequate resources to protect the data. Imaging variables can be requested as extracted summary values (.csv) or as minimally processed source files (DICOM). Raw images will also be available in 2024. Interested researchers can also search previously approved data requests. Specific request example. To access (or generate) the data specifically described in this resource, an investigator would request the following: Imaging variables: standard uptake ratio values calculated after partial volume correction for the PiB summary metric (amyloid), the FDG isthmus cingulate and inferior parietal region (hypometabolism) as well as MRI-derived intracranial volume and hippocampal volume (hippocampal atrophy) and cortical thickness summary measure (cortical atrophy). Demographic and descriptive variables: age, sex, race, ADAD mutation-carrying status, ADAD mutation type, clinical dementia rating, EYO, family ID and *APOE* carrier status. Additionally, referring to this paper would further streamline the data curation process.

## Code availability

The analysis scripts used to generate the images and statistical output in this paper are available from the Imaging Core Laboratory GitHub repository (https://github.com/benzinger-icl/DIAN_Imaging_Methods_2023). These scripts use openly accessible packages within the R (version 4.2.2) environment. The associated ‘read.me’ file also provides detailed instructions regarding how to use the provided code.

## the Dominantly Inherited Alzheimer Network

Nicole S. McKay^1^, Brian A. Gordon^1^, Russ C. Hornbeck^1^, Aylin Dincer^1^, Shaney Flores^1^, Sarah J. Keefe^1^, Nelly Joseph-Mathurin^1^, Clifford R. Jack^2^, Robert Koeppe^3^, Peter R. Millar^1^, Beau M. Ances^1^, Charles D. Chen^1^, Alisha Daniels^1^, Diana A. Hobbs^1^, Kelley Jackson^1^, Deborah Koudelis^1^, Parinaz Massoumzadeh^1^, Austin McCullough^1^, Michael L. Nickels^1^, Farzaneh Rahmani^1^, Laura Swisher^1^, Qing Wang^1^, Ricardo F. Allegri^4^, Sarah B. Berman^5^, Adam. M. Brickman^6^, William S. Brooks^7^, David M. Cash^8,9^, Jasmeer P. Chhatwal^10^, Gregory S. Day^11^, Martin R. Farlow^12^, Christian la Fougère^13^, Nick C. Fox^8,9^, Michael Fulham^15^, Bernardino Ghetti^12^, Neill Graff-Radford^11^, Takeshi Ikeuchi^16^, William Klunk^5^,Jae-Hong Lee^17^, Johannes Levin^18^, Ralph Martins^19^, Colin L. Masters^20^, Jonathan McConathy^21^, Hiroshi Mori^22^, James M. Noble^6^, Gerald Reischl^13^, Christopher Rowe^20^, Stephen Salloway^23^, Raquel Sanchez-Valle^24^, Peter R. Schofield^7,25^, Hiroyuki Shimada^22^, Mikio Shoji^26^, Yi Su^27^, Kazushi Suzuki^28^, Jonathan Vöglein^18,29^, Igor Yakushev^30^, Carlos Cruchaga^1^, Jason Hassenstab^1^, Celeste Karch^1^, Eric McDade^1^, Richard J. Perrin^1^, Chengjie Xiong^1^, John C. Morris^1^, Randall J. Bateman^1^ & Tammie L. S. Benzinger^1^

## References

[1] Fox NC & Petersen RC The G8 Dementia Research Summit—a starter for eight? Lancet 382, 1968–1969 (2013). 24332306 10.1016/S0140-6736(13)62426-5

[2] Mueller SG The Alzheimer’s disease neuroimaging initiative. Neuroimaging Clin. N. Am 15, 869–877 (2005). 16443497 10.1016/j.nic.2005.09.008 PMC2376747

[3] Ellis KA The Australian Imaging, Biomarkers and Lifestyle (AIBL) study of aging: methodology and baseline characteristics of 1112 individuals recruited for a longitudinal study of Alzheimer’s disease. Int. Psychogeriatr 21, 672–687 (2009). 19470201 10.1017/S1041610209009405

[4] Morris JC Developing an international network for Alzheimer research: the Dominantly Inherited Alzheimer Network. Clin. Investig. (Lond) 2, 975–984 (2012). 10.4155/cli.12.93 PMC3489185 23139856

[5] Bateman RJ Clinical and biomarker changes in dominantly inherited Alzheimer’s disease. N. Engl. J. Med 367, 795–804 (2012). 22784036 10.1056/NEJMoa1202753 PMC3474597

[6] Cruchaga C, Chakraverty S, Mayo K & Vallania FLM Rare variants in *APP, PSEN1* and *PSEN2* increase risk for AD in late-onset Alzheimer’s disease families. PLoS ONE 7, e31039 (2012). 22312439 10.1371/journal.pone.0031039 PMC3270040

[7] Hampel H. The amyloid-β pathway in Alzheimer’s disease. Mol. Psychiatry 26, 5481–5503 (2021). 34456336 10.1038/s41380-021-01249-0 PMC8758495

[8] Ryman DC Symptom onset in autosomal dominant Alzheimer disease: a systematic review and meta-analysis. Neurology 83, 253–260 (2014). 24928124 10.1212/WNL.0000000000000596 PMC4117367

[9] Chen CD Comparing amyloid-β plaque burden with antemortem PiB PET in autosomal dominant and late-onset Alzheimer disease. Acta Neuropathol. 142, 689–706 (2021). 34319442 10.1007/s00401-021-02342-y PMC8815340

[10] Chen CD Ante- and postmortem tau in autosomal dominant and late-onset Alzheimer’s disease. Ann. Clin. Transl. Neurol 7, 2475–2480 (2020). 33150749 10.1002/acn3.51237 PMC7732239

[11] Salloway S. A trial of gantenerumab or solanezumab in dominantly inherited Alzheimer’s disease. Nat. Med 27, 1187–1196 (2021). 34155411 10.1038/s41591-021-01369-8 PMC8988051

[12] Morris JC Autosomal dominant and sporadic late onset Alzheimer disease share a common in vivo pathophysiology. Brain 145, 3594–3607 (2022). 35580594 10.1093/brain/awac181 PMC9989348

[13] Benzinger TLS Regional variability of imaging biomarkers in autosomal dominant Alzheimer’s disease. Proc. Natl Acad. Sci. USA 110, E4502–E4509 (2013). 24194552 10.1073/pnas.1317918110 PMC3839740

[14] Gordon BA Tau PET in autosomal dominant Alzheimer’s disease: relationship with cognition, dementia and other biomarkers. Brain 142, 1063–1076 (2019). 30753379 10.1093/brain/awz019 PMC6439328

[15] Boerwinkle A. Comparison of amyloid accumulation between Down syndrome and autosomal-dominant Alzheimer disease. Alzheimers Dement. 18, e064684 (2022).

[16] Boerwinkle AH Comparison of amyloid burden in individuals with Down syndrome versus autosomal dominant Alzheimer’s disease: a cross-sectional study. Lancet Neurol. 22, 55–65 (2023). 36517172 10.1016/S1474-4422(22)00408-2 PMC9979840

[17] De Jonghe C. Flemish and Dutch mutations in amyloid β precursor protein have different effects on amyloid β secretion. Neurobiol. Dis 5, 281–286 (1998). 9848098 10.1006/nbdi.1998.0202

[18] Morris JC The Clinical Dementia Rating (CDR): current version and scoring rules. Neurology 43, 2412–2414 (1993). 10.1212/wnl.43.11.2412-a 8232972

[19] R Core Team. R: a language and environment for statistical computing. https://www.r-project.org/ (R Foundation for Statistical Computing, 2019).

[20] Folstein MF, Folstein SE & McHugh PR ‘Mini-mental state’: a practical method for grading the cognitive state of patients for the clinician. J. Psychiatr. Res 12, 189–198 (1975). 1202204 10.1016/0022-3956(75)90026-6

[21] Wechsler D. The psychometric tradition: developing the Wechsler Adult Intelligence Scale. Contemp. Educ. Psychol 6, 82–85 (1981).

[22] Wechsler D. Wechsler Memory Scale 3rd edn (Pearson, 1997).

[23] Goodglass H & Kaplan E Boston Diagnostic Aphasia Examination (Lea & Febiger, 1983).

[24] Su Y. Quantitative analysis of PiB-PET with FreeSurfer ROIs. PLoS ONE 8, e73377 (2013). 24223109 10.1371/journal.pone.0073377 PMC3819320

[25] Fischl B. Whole brain segmentation: automated labeling of neuroanatomical structures in the human brain. Neuron 33, 341–355 (2002). 11832223 10.1016/s0896-6273(02)00569-x

[26] Dincer A. Comparing cortical signatures of atrophy between late-onset and autosomal dominant Alzheimer disease. Neuroimage Clin. 28, 102491 (2020). 33395982 10.1016/j.nicl.2020.102491 PMC7689410

[27] Chhatwal JP Variant-dependent heterogeneity in amyloid β burden in autosomal dominant Alzheimer’s disease: cross-sectional and longitudinal analyses of an observational study. Lancet Neurol. 21, 140–152 (2022). 35065037 10.1016/S1474-4422(21)00375-6 PMC8956209

[28] Greenberg SM Cerebral amyloid angiopathy and Alzheimer disease—one peptide, two pathways. Nat. Rev. Neurol 16, 30–42 (2020). 31827267 10.1038/s41582-019-0281-2 PMC7268202

[29] Horien C. A hitchhiker’s guide to working with large, open-source neuroimaging datasets. Nat. Hum. Behav 5, 185–193 (2021). 33288916 10.1038/s41562-020-01005-4 PMC7992920

[30] Goyal MS Persistent metabolic youth in the aging female brain. Proc. Natl Acad. Sci. USA 116, 3251–3255 (2019). 30718410 10.1073/pnas.1815917116 PMC6386682

[31] Koenig LN Select atrophied regions in Alzheimer disease (SARA): an improved volumetric model for identifying Alzheimer disease dementia. Neuroimage Clin. 26, 102248 (2020). 32334404 10.1016/j.nicl.2020.102248 PMC7182765

[32] Millar PR Predicting brain age from functional connectivity in symptomatic and preclinical Alzheimer disease. Neuroimage 256, 119228 (2022). 35452806 10.1016/j.neuroimage.2022.119228 PMC9178744

[33] Millar PR Multimodal brain age estimates relate to Alzheimer disease biomarkers and cognition in early stages: a cross-sectional observational study. Elife 12, e81869 (2023). 36607335 10.7554/eLife.81869 PMC9988262

[34] Bethlehem RAI Brain charts for the human lifespan. Nature 604, 525–533 (2022). 35388223 10.1038/s41586-022-04554-y PMC9021021

[35] Preische O. Serum neurofilament dynamics predicts neurodegeneration and clinical progression in presymptomatic Alzheimer’s disease. Nat. Med 25, 277–283 (2019). 30664784 10.1038/s41591-018-0304-3 PMC6367005

[36] Cash DM The pattern of atrophy in familial Alzheimer disease: volumetric MRI results from the DIAN study. Neurology 81, 1425–1433 (2013). 24049139 10.1212/WNL.0b013e3182a841c6 PMC3806583

[37] Gordon BA Spatial patterns of neuroimaging biomarker change in individuals from families with autosomal dominant Alzheimer’s disease: a longitudinal study. Lancet Neurol. 17, 241–250 (2018). 29397305 10.1016/S1474-4422(18)30028-0 PMC5816717

[38] Lee S. White matter hyperintensities and the mediating role of cerebral amyloid angiopathy in dominantly-inherited Alzheimer’s disease. PLoS ONE 13, e0195838 (2018). 29742105 10.1371/journal.pone.0195838 PMC5942789

[39] Lee S. White matter hyperintensities are a core feature of Alzheimer’s disease: evidence from the dominantly inherited Alzheimer network. Ann. Neurol 79, 929–939 (2016). 27016429 10.1002/ana.24647 PMC4884146

[40] Joseph-Mathurin N. Longitudinal accumulation of cerebral microhemorrhages in dominantly inherited Alzheimer disease. Neurology 96, e1632–e1645 (2021). 33495373 10.1212/WNL.0000000000011542 PMC8032370

[41] Cairns NJ Neuropathologic assessment of participants in two multi-center longitudinal observational studies: the Alzheimer Disease Neuroimaging Initiative (ADNI) and the Dominantly Inherited Alzheimer Network (DIAN). Neuropathology 35, 390–400 (2015). 25964057 10.1111/neup.12205 PMC4521391

[42] Chhatwal JP Preferential degradation of cognitive networks differentiates Alzheimer’s disease from ageing. Brain 141, 1486–1500 (2018). 29522171 10.1093/brain/awy053 PMC5917745

[43] Chhatwal JP Impaired default network functional connectivity in autosomal dominant Alzheimer disease. Neurology 81, 736–744 (2013). 23884042 10.1212/WNL.0b013e3182a1aafe PMC3776464

[44] Franzmeier N. Left frontal hub connectivity delays cognitive impairment in autosomal-dominant and sporadic Alzheimer’s disease. Brain 141, 1186–1200 (2018). 29462334 10.1093/brain/awy008 PMC5888938

[45] Smith R. Resting-state functional connectivity disruption as a pathological biomarker in autosomal dominant Alzheimer disease. Brain Connect. 11, 239–249 (2021). 33430685 10.1089/brain.2020.0808 PMC8182476

[46] Mills SM Preclinical trials in autosomal dominant AD: implementation of the DIAN-TU trial. Rev. Neurol 169, 737–743 (2013). 24016464 10.1016/j.neurol.2013.07.017 PMC3880800

[47] Schultz SA Serum neurofilament light chain levels are associated with white matter integrity in autosomal dominant Alzheimer’s disease. Neurobiol. Dis 142, 104960 (2020). 32522711 10.1016/j.nbd.2020.104960 PMC7363568

[48] Karch CM Human fibroblast and stem cell resource from the Dominantly Inherited Alzheimer Network. Alzheimers Res. Ther 10, 69 (2018). 30045758 10.1186/s13195-018-0400-0 PMC6060509

[49] Fagan AM Longitudinal change in CSF biomarkers in autosomal-dominant Alzheimer’s disease. Sci. Transl. Med 6, 226ra30 (2014). 10.1126/scitranslmed.3007901 PMC4038930 24598588

[50] Kruggel F, Turner J, Muftuler LT & Alzheimer’s Disease Neuroimaging Initiative. Impact of scanner hardware and imaging protocol on image quality and compartment volume precision in the ADNI cohort. Neuroimage 49, 2123–2133 (2010). 19913626 10.1016/j.neuroimage.2009.11.006 PMC2951115

[51] Beer JC Longitudinal ComBat: a method for harmonizing longitudinal multi-scanner imaging data. Neuroimage 220, 117129 (2020). 32640273 10.1016/j.neuroimage.2020.117129 PMC7605103

[52] Jack CR Jr The Alzheimer’s Disease Neuroimaging Initiative (ADNI): MRI methods. J. Magn. Reson. Imaging 27, 685–691 (2008). 18302232 10.1002/jmri.21049 PMC2544629

[53] Jagust WJ The Alzheimer’s Disease Neuroimaging Initiative 2 PET Core: 2015. Alzheimers Dement. 11, 757–771 (2015). 26194311 10.1016/j.jalz.2015.05.001 PMC4510459

[54] Ringman JM Neuropathology of autosomal dominant Alzheimer disease in the National Alzheimer Coordinating Center Database. J. Neuropathol. Exp. Neurol 75, 284–290 (2016). 26888304 10.1093/jnen/nlv028 PMC4934612

[55] Vermunt L. Single-subject grey matter network trajectories over the disease course of autosomal dominant Alzheimer’s disease. Brain Commun. 2, fcaa102 (2020). 32954344 10.1093/braincomms/fcaa102 PMC7475695

[56] Mori S & Zhang J Principles of diffusion tensor imaging and its applications to basic neuroscience research. Neuron 51, 527–539 (2006). 16950152 10.1016/j.neuron.2006.08.012

[57] Pierpaoli C & Basser PJ Toward a quantitative assessment of diffusion anisotropy. Magn. Reson. Med 36, 893–906 (1996). 8946355 10.1002/mrm.1910360612

[58] Wang Q. Quantification of white matter cellularity and damage in preclinical and early symptomatic Alzheimer’s disease. Neuroimage Clin. 22, 101767 (2019). 30901713 10.1016/j.nicl.2019.101767 PMC6428957

[59] Grimmer T. White matter hyperintensities predict amyloid increase in Alzheimer’s disease. Neurobiol. Aging 33, 2766–2773 (2012). 22410648 10.1016/j.neurobiolaging.2012.01.016

[60] Chavhan GB, Babyn PS, Thomas B, Shroff MM & Haacke EM Principles, techniques, and applications of T2*-based MR imaging and its special applications. Radiographics 29, 1433–1449 (2009). 19755604 10.1148/rg.295095034 PMC2799958

[61] Graff-Radford J. White matter hyperintensities: relationship to amyloid and tau burden. Brain 142, 2483–2491 (2019). 31199475 10.1093/brain/awz162 PMC6658846

[62] Soosman SK Widespread white matter and conduction defects in *PSEN1*-related spastic paraparesis. Neurobiol. Aging 47, 201–209 (2016). 27614114 10.1016/j.neurobiolaging.2016.07.030 PMC5075491

[63] Johnson NA Pattern of cerebral hypoperfusion in Alzheimer disease and mild cognitive impairment measured with arterial spin-labeling MR imaging: initial experience. Radiology 234, 851–859 (2005). 15734937 10.1148/radiol.2343040197 PMC1851934

[64] Takahashi H. Clinical application of 3D arterial spin-labeled brain perfusion imaging for Alzheimer disease: comparison with brain perfusion SPECT. AJNR Am. J. Neuroradiol 35, 906–911 (2014). 24263694 10.3174/ajnr.A3780 PMC7964532

[65] Du AT Hypoperfusion in frontotemporal dementia and Alzheimer disease by arterial spin labeling MRI. Neurology 67, 1215–1220 (2006). 17030755 10.1212/01.wnl.0000238163.71349.78 PMC1779761

[66] Li Y. ASL-MRICloud: an online tool for the processing of ASL MRI data. NMR Biomed. 32, e4051 (2019). 30588671 10.1002/nbm.4051 PMC6324946

[67] McDade E. Cerebral perfusion alterations and cerebral amyloid in autosomal dominant Alzheimer disease. Neurology 83, 710–717 (2014). 25031286 10.1212/WNL.0000000000000721 PMC4150128

[68] Biswal B & Zerrin Yetkin F Functional connectivity in the motor cortex of resting human brain using echo-planar MRI. Magn. Reson. Med 34, 537–541 (1995). 8524021 10.1002/mrm.1910340409

[69] Buckner RL Molecular, structural, and functional characterization of Alzheimer’s disease: evidence for a relationship between default activity, amyloid, and memory. J. Neurosci 25, 7709–7717 (2005). 16120771 10.1523/JNEUROSCI.2177-05.2005 PMC6725245

[70] Jack CR Jr Tracking pathophysiological processes in Alzheimer’s disease: an updated hypothetical model of dynamic biomarkers. Lancet Neurol. 12, 207–216 (2013). 23332364 10.1016/S1474-4422(12)70291-0 PMC3622225

[71] Su Y. Partial volume correction in quantitative amyloid imaging. Neuroimage 107, 55–64 (2015). 25485714 10.1016/j.neuroimage.2014.11.058 PMC4300252

[72] Su Y. Quantitative amyloid imaging in autosomal dominant Alzheimer’s disease: results from the DIAN Study Group. PLoS ONE 11, e0152082 (2016). 27010959 10.1371/journal.pone.0152082 PMC4807073

[73] Su Y. Utilizing the centiloid scale in cross-sectional and longitudinal PiB PET studies. Neuroimage Clin. 19, 406–416 (2018). 30035025 10.1016/j.nicl.2018.04.022 PMC6051499

[74] Klunk WE The Centiloid Project: standardizing quantitative amyloid plaque estimation by PET. Alzheimers Dement. 11, 1-15. e1–1-15e4 (2015). 25443857 10.1016/j.jalz.2014.07.003 PMC4300247

[75] Buckner LB, Head D, Parker P, Fotenos AF & Marcus D A unified approach for morphometric and functional data analysis in young, old, and demented adults using automated atlas-based head size normalization Neuroimage 23, 724–738 (2004). 15488422 10.1016/j.neuroimage.2004.06.018

