# Supplementary material for: Positron emission tomography and magnetic resonance imaging methods and datasets within the Dominantly Inherited Alzheimer Network (DIAN)
Source: Nat Neurosci. Author manuscript; Available in PMC 2023 Sep 27. (PMC10400428; doi:10.1038/s41593-023-01359-8)
Supplement: 1 [file NIHMS1917384-supplement-1.pdf]

nature portfolio

Corresponding author(s): Tammie L.S. Benzinger

Last updated by author(s): May 13, 2023

Reporting Summary

Nature Portfolio wishes to improve the reproducibility of the work that we publish. This form provides structure for consistency and transparency in reporting. For further information on Nature Portfolio policies, see our Editorial Policies and the Editorial Policy Checklist.

Statistics

For all statistical analyses, confirm that the following items are present in the figure legend, table legend, main text, or Methods section.

n/a	Confirmed	
☐	☒	The exact sample size (*n*) for each experimental group/condition, given as a discrete number and unit of measurement
☐	☒	A statement on whether measurements were taken from distinct samples or whether the same sample was measured repeatedly
☐	☒	The statistical test(s) used AND whether they are one- or two-sided<br>*Only common tests should be described solely by name; describe more complex techniques in the Methods section.*
☐	☒	A description of all covariates tested
☐	☒	A description of any assumptions or corrections, such as tests of normality and adjustment for multiple comparisons
☐	☒	A full description of the statistical parameters including central tendency (e.g. means) or other basic estimates (e.g. regression coefficient) AND variation (e.g. standard deviation) or associated estimates of uncertainty (e.g. confidence intervals)
☐	☒	For null hypothesis testing, the test statistic (e.g. *F*, *t*, *r*) with confidence intervals, effect sizes, degrees of freedom and *P* value noted<br>*Give P values as exact values whenever suitable.*
☒	☐	For Bayesian analysis, information on the choice of priors and Markov chain Monte Carlo settings
☒	☐	For hierarchical and complex designs, identification of the appropriate level for tests and full reporting of outcomes
☐	☒	Estimates of effect sizes (e.g. Cohen's *d*, Pearson's *r*), indicating how they were calculated

Our web collection on statistics for biologists contains articles on many of the points above.

Software and code

Policy information about availability of computer code

Data collection	No software or code was used to collect data for this manuscript.
Data analysis	Prior to analyses, data were segmented using FreeSurfer (version 5.3) and the PET unified Pipieline (PUP) https://github.com/ysu001/PUP. To run our analyses, we used the extracted output of these processing steps, which can be requested as extracted summary imaging data. All analyses were conducted within the R environment (version 4.2.2). An R script is available through github that can reproduce almost every part of the manuscript: https://github.com/benzinger-icl/DIAN_Imaging_Methods_2023. To prevent unblinding of participants, we will not be able to release the portion of the code creating the plot that depicts "site contributions" as some sites have very few individuals and their associated information would un-blind them. However, if this modification to the code is an issue, we could de-identify the site data so it can not be linked back to individuals, so that this small portion of code could also be released alongside the manuscript. A read me file is also included with the code script file to outline the exact variables that should be requested as part of the data request, if individuals are wanting to replicate the included analyses.

For manuscripts utilizing custom algorithms or software that are central to the research but not yet described in published literature, software must be made available to editors and reviewers. We strongly encourage code deposition in a community repository (e.g. GitHub). See the Nature Portfolio guidelines for submitting code & software for further information.

Data

Policy information about availability of data

All manuscripts must include a data availability statement. This statement should provide the following information, where applicable:

- Accession codes, unique identifiers, or web links for publicly available datasets
- A description of any restrictions on data availability
- For clinical datasets or third party data, please ensure that the statement adheres to our policy

All datasets described within the current resource manuscript are freely available upon the completion of a Dominantly Inherited Alzheimer Network Data Request, (https://dian.wustl.edu/our-research/for-investigators/dian-observational-study-investigator-resources/data-request-terms-and-instructions/). Imaging data is available as extracted averages from FreeSurfer derived regions of interest (.xlsx), or identity-stripped source files (DICOM). Specifically, requesting the following data points from DIAN data release 15: MRI: cortical thickness signature, intracranial volume, hippocampal volume; PET partial volume corrected SUVRs for: FDG-isthmus, FDG-inferior parietal, PiB-PET summary regions; Demographics: Age, Sex, Education; Clinical/Cognition: CDR, MMSE, WAIS, delayed logical memory, Animal naming task, Boston naming task; Genetics: family ID, mutation carrying status, ADAD mutation type, age of expected symptom onset.

Research involving human participants, their data, or biological material

Policy information about studies with human participants or human data. See also policy information about sex, gender (identity/presentation), and sexual orientation and race, ethnicity and racism.

Reporting on sex and gender	In our demographic breakdown of the DIAN-OBS data, we have included counts of males and females to describe the distributions of biological sex within our cohorts. Through use of Chi-Square tests, we have shown that the distributions of sex do not differ across our three groups, and for simplicity we do not investigate specific sex-related effects further. We have included a more in-depth demographics visualization (Figure 1), which allows readers to visualize easily the proportion of females and males in each group (non-carriers, asymptomatic mutation carriers, and symptomatic mutation carriers), as well as the relative distribution of ages for each sex.
Reporting on race, ethnicity, or other socially relevant groupings	The DIAN-OBS does collect data relevant to race and ethnicity, however, we do not use it within this manuscript to make any inferences. The DIAN Cohort is predominantly White and not Hispanic. In our Extended Data we provide a visualization that shows the relative proportions of individuals who self-identify as: White, Asian, Aboriginal Australian or Torres Strait Islander, Latin American, Middle Eastern, Black or African American, Hispanic, Native Hawaiian or Pacific Islander, North African, Mexican, European, American Indian or Alaska Native. We also show a breakdown of secondary and tertiary self-identified race within this cohort.
Population characteristics	We have included several major descriptive variables to give an overview of the characteristics of this population. We include age, sex, handedness, years of education, race, time to expected symptom onset (EYO), cognitive impairment status (CDR), their baseline cognitive accuracy (a cognitive composite), their baseline MMSE scores of clinical impairment, and some basic genetic information. We explicitly show their APOE status, their ADAD mutation status, and for within those with PSEN1 mutations, we report whether their mutation falls pre- or post- codon 200, allowing researchers to get an idea of how many individuals can be utilized for replications of recent PSEN1 studies showing codon position changes phenotypic expression in ADAD mutation carriers. Important covariates that should be included in analyses include family ID, to account for non-independent data points from members of the same family. It is also often important to include age, sex, and EYO as covariates, depending on the question of interest. Within our examples, we have used family ID, age, sex, and education as covariates.
Recruitment	Participants were recruited through the various DIAN collaboration sites (21 across the globe), as well as through broader efforts such as: http://dian-info.org/, http://www.alzforum.org/new/detail.asp?id=1967, http://www.alz.org/trialmatch and http://www.dianexpandedregistry.org/. As individuals come from families with known histories of ADAD, there is a strong possibility for selection bias, or prior knowledge of ADAD upon entering the study. To remove some forms of this bias, participants are not required to learn their mutation carrying status, and study coordinators running assessments are blinded to their mutation carrying and CDR status at time of testing.
Ethics oversight	Procedures were approved by Washington University Human Research Protection Office, the central IRB for the DIAN study. However, local IRBs of the participating sites also approved of all study procedures.

Note that full information on the approval of the study protocol must also be provided in the manuscript.

Field-specific reporting

Please select the one below that is the best fit for your research. If you are not sure, read the appropriate sections before making your selection.

☒ Life sciences ☐ Behavioural & social sciences ☐ Ecological, evolutionary & environmental sciences

For a reference copy of the document with all sections, see nature.com/documents/nr-reporting-summary-flat.pdf

Life sciences study design

All studies must disclose on these points even when the disclosure is negative.

Sample size	Sample size was determined based on the number of individuals within the DIAN study who underwent neuroimaging sessions. Analyses with fewer individuals represent modalities of neuroimaging that had some data drop out due to failure of quality control processes (outlined within the methods section) or because the modality was added during a more recent update to the imaging protocols. In all cases, all DIAN data release #15 data was included.
Data exclusions	Only images that did not pass quality control measures (prior to summary data extraction) were excluded. These procedures are outlined within the methods section of the manuscript.
Replication	We have deposited our code here: https://github.com/benzinger-icl/DIAN_Imaging_Methods_2023 this can be used by researchers who request DIAN-OBS data to replicate the analyses described within. This code will also allow researchers to alter data analyses to match their individual project needs. Further, all analyses included within are nature portfolio | reporting summary March 2021 designed to provide descriptive information for those wishing to use this imaging data resource. In all cases, the data adhered to expected patterns consistent with literature that has previously used the DIAN-OBS data for analyses (and citations are provided within).
Randomization	Individuals were grouped into: mutation carriers and non carriers based on the results of their genetic data. A further split was conducted to consider their cognitive impairment status, where those who scored greater than zero on the CDR scale, were considered cognitive impaired. Combining these two groupings results in four distinct groups: unimpaired non-carriers, impaired non-carriers, asymptomatic mutation carriers, and symptomatic mutation carriers. There are only four impaired non-carriers, so we did not consider them separately in these analyses.
Blinding	Those who were directly testing the individuals remained blinded to their impairment and mutation-carrying status.

Reporting for specific materials, systems and methods

We require information from authors about some types of materials, experimental systems and methods used in many studies. Here, indicate whether each material, system or method listed is relevant to your study. If you are not sure if a list item applies to your research, read the appropriate section before selecting a response.

Materials & experimental systems

n/a	Involved in the study	
☒	☐	Antibodies
☒	☐	Eukaryotic cell lines
☒	☐	Palaeontology and archaeology
☒	☐	Animals and other organisms
☒	☐	Clinical data
☒	☐	Dual use research of concern
☒	☐	Plants

Methods

n/a	Involved in the study	
☒	☐	ChIP-seq
☒	☐	Flow cytometry
☐	☒	MRI-based neuroimaging

Magnetic resonance imaging

Experimental design

Design type	Resting state
Design specifications	rsMRI was acquired in the axial orientation with a multiband factor of 1, while participants were asked to keep their eyes open in order to ensure they did not fall asleep.
Behavioral performance measures	Behavioural performance was not recorded during the MRI session, all cognitive and clinical assessments were conducted in a separate session to imaging acquisition.

Acquisition

Imaging type(s)	functional, structural, perfusion, diffusion, amyloid-PET, FDG-PET
Field strength	3T
Sequence & imaging parameters	All imaging data was acquired using the ADNI MRI and PET protocols.<br>rsMRI. The majority (n=394) of rsMRI scans were acquired using: TE=30ms, TR=2230ms, flip angle=80 degrees, acquisition matrix=64x58x36, voxel size=3.3x3.3x3.3mm. For the remaining rsMRI scans, we provide a table explicitly outlining the values for each of these parameters. Eyes were open.<br>T1 MPRAGE. TE=2.95ms, TR=2300ms, TI=900ms, FOV=270mm, flip angle=9 degrees, number of slices=225, voxel size=1.1 x 1.1 x 1.2 mm3, GRAPPA acceleration factor=2.

ASL. Depending on location (and therefore scanner availability and parameter constraints), ASL was run using 2D- or 3D PASL (PICORE, STAR, FAIR tags). Resolution was either 3.2 x 3.2 x 4 (for 2D sequences) or 1.9 x 1.9 x 4.5 (for 3D sequences). TR was 3400 (2D) or 4000 (3D), TE was 13 (2D) or 21.8 (3D), TI1 was 700 (2D) or 800 (3D) and TI was 1900 (2D) or 2000 (3D).
T2 FLAIR. Acquired axially using: TE=91ms, TR=9000ms, TI=2500ms, FOV=220mm, flip angle=150 degrees, slices=35, voxel size=0.9 x 0.9 x 5mm, acceleration factor=2.
T2 GRE. STAR/GRE were acquired using: TE=20ms, TR=650ms, FOV=200mm, Flip angle=20 degrees, slices=44, voxel size=0.8 x 0.8 x 4mm.
Amyloid-PET: Using 15 mCi PiB tracer, data was acquired for 70 mins, with the final 30 mins being used for data analyses.
FDG-PET. Using 5 mCi FDG tracer, data was acquired for 30 minutes following a delay of 30 mins after injection.

Area of acquisition: Whole-brain

Diffusion MRI ☒ Used ☐ Not used

Parameters: The DIAN-DBSI sequence comprises three diffusion sequence sessions with the Siemens built-in 6, 10, and 12 diffusion vectors, respectively. Multiple b-values were implemented in each session. The maximal b-values for each session are 2000, 1500, and 1000 s/mm2, respectively. By combining all three sessions, a total of 28 unique directions were acquired, with 66 unique diffusion weightings. For each run, there was one volume with no diffusion weighting (b=0 s/mm2) accounting for the remaining volumes.

Preprocessing

Preprocessing software: Software versions are stated within the manuscript; FreeSurfer (5.3 - HCP patch) and R (4.2.2), are two freely available software programs. Citations for each of these are noted within the manuscript. There is also a citation, and github link, to the freely available PET-Unified-Processing pipeline (PUP) provided within the manuscript which is used for processing the PET data; https://github.com/ysu001/PUP

Normalization: Freesurfer's recon-all flag includes transformation to Talairach space and aseg atlas

Normalization template: Talairach

Noise and artifact removal: Freesurfer's recon-all function employs motion correction, removal of non-brain structures using a watershed deformation procedure, intensity normalization, these specific descriptions within the manuscript include citations. Furthermore, quality control technicians screen images prior to preprocessing to ensure any major deviations or artifacts are caught and they will request sites to re-scan individuals if necessary. Finally, further quality control procedures occur at the end of the Freesurfer recon-all preprocessing to ensure remaining artifacts or errors are removed. These processing technicians will run error checks and subsequent reprocessing of the data up to three times before images are considered to have failed.

Volume censoring: We do not censor volumes, we only remove data from the data release that have freesurfer errors that cannot be rectified.

Statistical modeling & inference

Model type and settings: The majority of the models within were ANCOVAs assessing group differences between three groups (while controlling for non-independent data arising from within-data family structure). Categorical variables were assessed using chi-square tests. All tests within were two-sided.

Effect(s) tested: Within, we demonstrate descriptive details of the differences between unimpaired non-carriers, asymptomatic mutationcarriers, and symptomatic mutation-carriers. We provide test statistics, confidence intervals, effect sizes, and p-values for interpretation, as well as inclusion of means and standard errors during interpretation of these statistics.

Specify type of analysis: ☐ Whole brain ☐ ROI-based ☒ Both

Anatomical location(s): Freesurfer defined anatomical locations were used for hippocampal analyses. The summary measures were investigated using the other neuroimaging biomarkers were previously validated in prior work, which is described and cited within, but also the result of analyses performed on freesurfer derived segmentations.

Statistic type for inference (See Eklund et al. 2016): Extracted averages are used for these analyses

Correction: Each analysis was testing a general, and distinct, hypothesis, therefore corrections were not used for the specific analyses implemented within. However, in follow-up pairwise comparisons, bonferroni corrections were made.

Models & analysis

n/a	Involved in the study	
☒	☐	Functional and/or effective connectivity
☒	☐	Graph analysis
☒	☐	Multivariate modeling or predictive analysis
